# Mining raw plant transcriptomic data for new cyclopeptide alkaloids

**DOI:** 10.3762/bjoc.20.138

**Published:** 2024-07-11

**Authors:** Draco Kriger, Michael A Pasquale, Brigitte G Ampolini, Jonathan R Chekan

**Affiliations:** 1 Department of Chemistry and Biochemistry, University of North Carolina at Greensboro, Greensboro, NC, USA

**Keywords:** burpitides, natural products, plants, RiPPs, transcriptome mining

## Abstract

In recent years, genome and transcriptome mining have dramatically expanded the rate of discovering diverse natural products from bacteria and fungi. In plants, this approach is often more limited due to the lack of available annotated genomes and transcriptomes combined with a less consistent clustering of biosynthetic genes. The recently identified burpitide class of ribosomally synthesized and post-translationally modified peptide (RiPP) natural products offer a valuable opportunity for bioinformatics-guided discovery in plants due to their short biosynthetic pathways and gene encoded substrates. Using a high-throughput approach to assemble and analyze 700 publicly available raw transcriptomic data sets, we uncover the potential distribution of split burpitide precursor peptides in Streptophyta. Metabolomic analysis of target plants confirms our bioinformatic predictions of new cyclopeptide alkaloids from both known and new sources.

## Introduction

**Figure 1:**
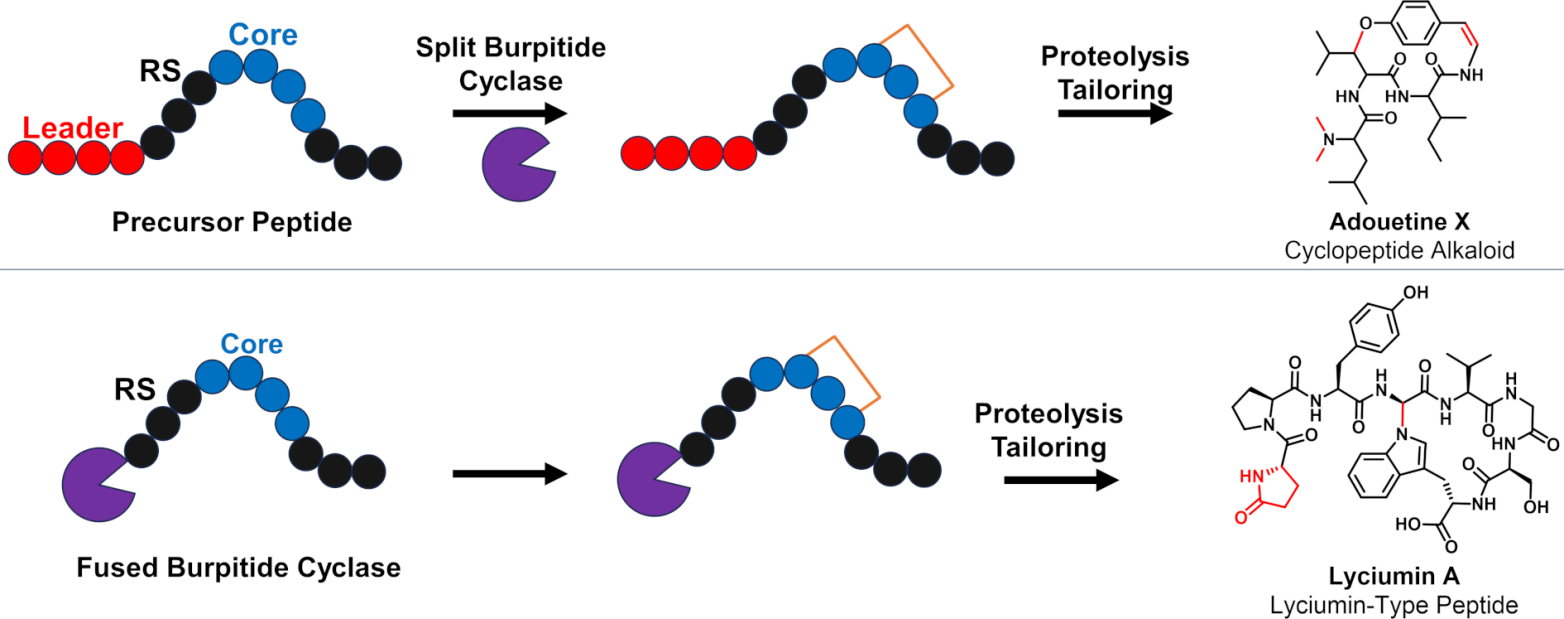
Biosynthetic scheme for the formation of burpitides using a split (top) or fused (bottom) pathway. RS is recognition sequence. Peptide modifications are shown in red.

Plants are prolific producers of cyclic peptide natural products, making 1000s of different molecules [1]. While the orbitide [2] and cyclotide [3] classes of peptides are well known, it has been recently discovered that a new class of molecules called burpitides are also prevalent in plants [4]. Like all known plant peptides, burpitides fall under the ribosomally synthesized and post-translationally modified peptides (RiPPs) superclass of natural products (Figure 1). The typical pathway for a RiPP starts with a precursor peptide made by the ribosome that undergoes post-translational modifications by specific tailoring enzymes [5–6]. This precursor peptide substrate can be subdivided into multiple segments including 1) an N-terminal leader or recognition sequence used for binding by the tailoring enzymes and 2) a core peptide that is targeted for modification by the biosynthetic enzymes. Ultimately proteolysis releases the modified core peptide as the mature RiPP natural product [5–6].

In the case of the newly described burpitide family of RiPPs, the defining feature is the presence of amino acid side-chain crosslinks installed by a copper-dependent burpitide cyclase [4]. These RiPP natural products encompass a wide range of structural scaffolds including cyclopeptide alkaloids that contain a phenolic ether-linkage and the lyciumin-type peptides that are composed of a crosslink between the Trp-indole-N and the carbon in another amino acid side chain or peptide backbone (Figure 1). Recent enzymatic reconstitution has demonstrated that the burpitide cyclases can function either autocatalytically (fused) or as traditional stand-alone proteins with separate free peptide substrates (split) [4,7–11]. When fully matured, burpitides possess a wide range of bioactivities including analgesic, sedative, and cytotoxic [12–14].

In order to identify new potential burpitides, we searched the NCBI Sequence Read Archive (SRA) for Streptophyta (all land plants and most green algae) transcriptomes that could be assembled and examined with a custom hidden Markov model (HMM) for split burpitide precursor peptides. Using this approach, we assembled 700 transcriptomes and used predicted precursor peptide diversity for target discovery of new cyclopeptide alkaloids. Additionally, metabolomic analysis revealed the tissue distribution of these molecules across select members of the Rubiaceae and Rhamnaceae families. Ultimately, our results lay the groundwork for the rapid discovery of new burpitides across diverse plant species.

## Results and Discussion

### Assembly and analysis of 700 public transcriptomes

In order to efficiently download, assemble, and analyze raw SRA data in a high throughput manner, we created an automated pipeline. Raw Illumina sequencing data were downloaded using the NCBI SRA Toolkit, the transcriptome was then assembled with rnaSPAdes [15], and the open reading frames were detected with TransDecoder [16–17]. To search for putative precursor peptides for split burpitide pathways, we developed a custom HMM (see Supporting Information File 1, Figure S1). This approach gave us the speed, sensitivity, and customization needed to identify diverse sequences. We targeted a diversity of plants from every available order in Streptophyta for a total of 700 transcriptomes from 647 species (Table in Supporting Information File 2).

**Figure 2:**
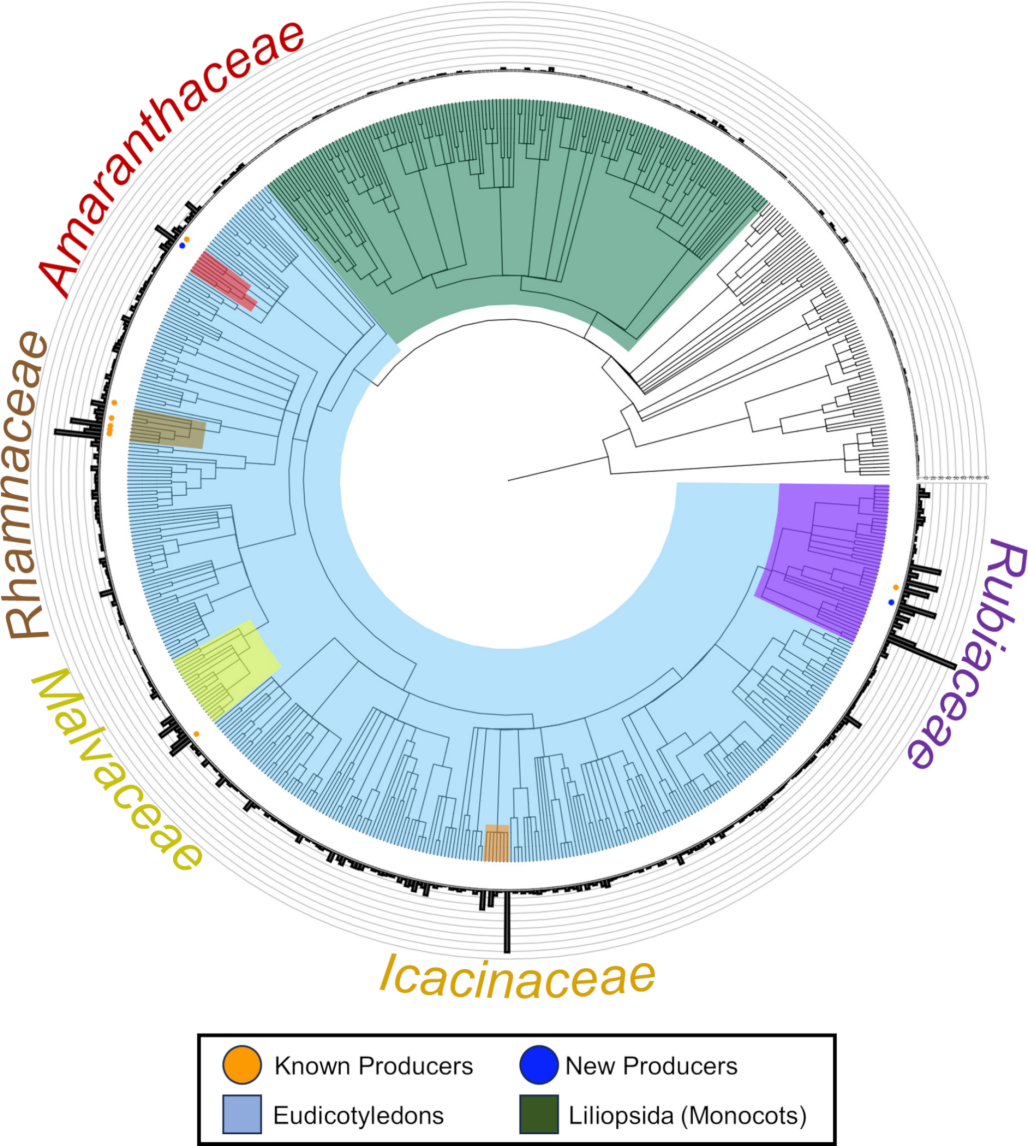
Cladogram made from 647 plant species surveyed. The bar graphs show the number of average unique transcripts from each plant species. Producers are indicated with an orange or blue dot.

Potential split burpitide precursor peptide transcripts were found in 66% of the assemblies using the custom HMM. However, due to the only recent discovery of split burpitides, the rules that dictate the presence of a candidate precursor peptide and observation of a corresponding small molecule are unclear. For example, precursors rich in S/TxY core motifs are prevalent in nature, but do not correspond to a known molecule [7]. Therefore, we sought to rapidly identify the most promising candidates. This presented a challenge as the publicly available data was generated using different protocols, from different plant tissues, and with different levels of sequencing depth. Ultimately, we reasoned that the best candidate plants for prolific production of burpitides should have numerous unique precursor peptide transcripts which may arise from multiple gene copies, multiple core sequences, or multiple RNA splicing events. Therefore, we counted the number of translated transcripts from each assembly that scored better than an E-value of 0.1 using our custom precursor peptide HMM. To select for only the split precursor peptides, we removed any transcript that had contained a BURP domain as determined by the public HMM model (PFAM 03181) with an E-value of 0.1 inclusion threshold. The BURP domain itself is named after the four founding members BNM2, USP-like, RD22, and PG1β, which were bioinformatically described in 1998 [18]. This domain is typically around 300 amino acids in length and has a conserved CHX_10_CHX_25_-_27_CHX_25_-_26_CH motif which comprises the active site of the burpitide cyclase. The resulting number of stand-alone transcripts from this filtering step were mapped onto a cladogram of the 647 species we surveyed (Figure 2 and Supporting Information File 3). For species with multiple transcriptomes, the number of precursor peptides was averaged (Table in Supporting Information File 2). We further mapped known producers with a corresponding precursor peptide onto the cladogram (orange dots). Using a cut-off of 30 unique transcripts, there were five clear phylogenetic hot spots for the production of burpitides. These families were examined in detail for their composition of core peptide sequences.

#### Rhamnaceae family

**Figure 3:**
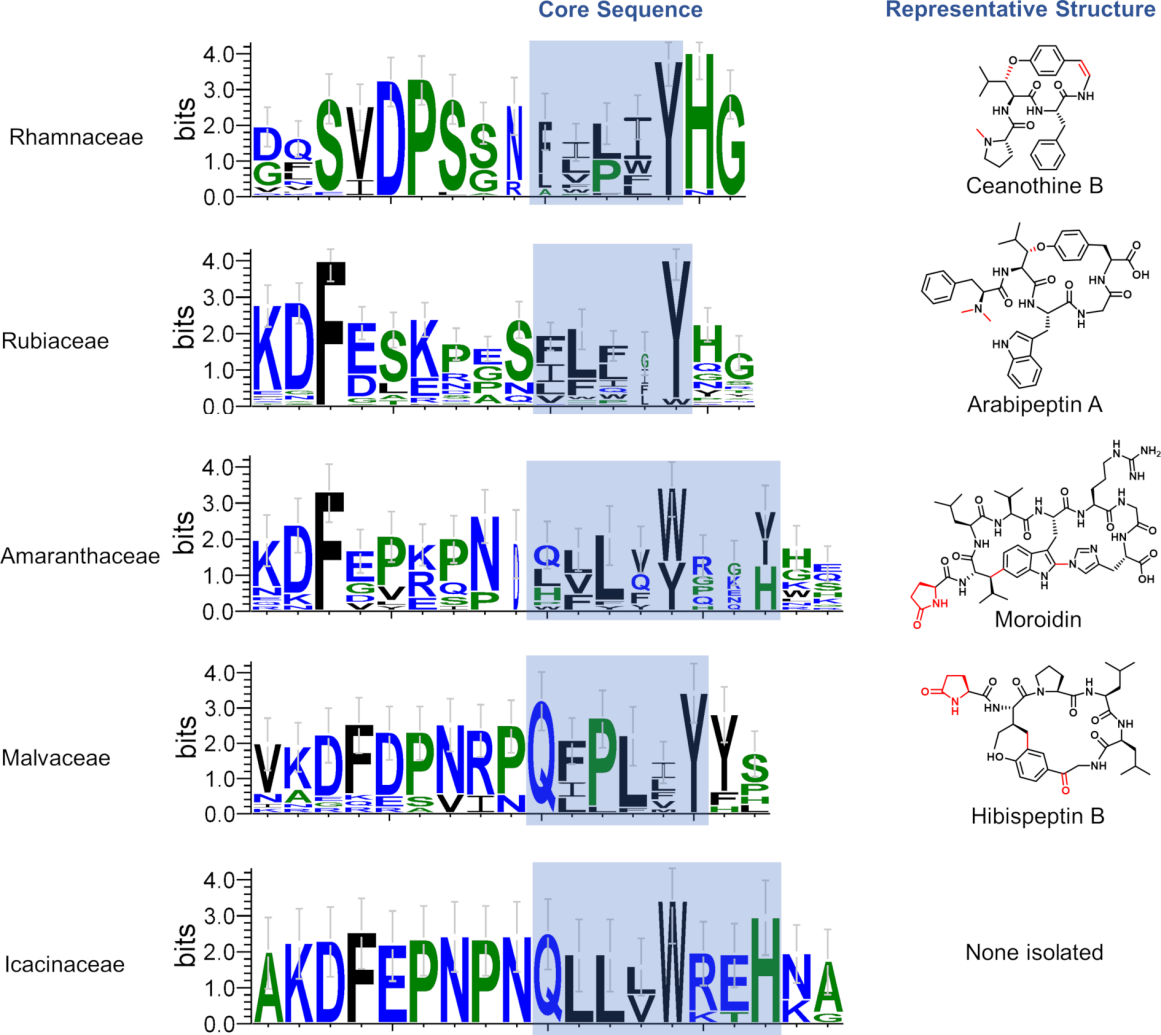
Weblogos generated with aligned recognition and core sequences from the six different families discussed. Up to three unique sequences were taken from each assembly. See Supporting Information File 2 for exact sequences identified and used. The highlighted region is the predicted core peptide motif.

The Rhamnaceae family is amongst the most iconic producers of cyclopeptide alkaloids, containing both *Ziziphus* and *Ceanothus* genera. In addition to the subclass defining Tyr-phenol-O to carbon linkage, the cyclopeptide alkaloids of this family are typically oxidatively decarboxylated and *N*-methylated (Figure 1, adouetine X and Figure 3, ceanothine B) [19–21]. Our transcriptome analysis supports the prevalence of cyclopeptide alkaloids in Rhamnaceae with multiple species transcribing 20+ potential split precursor peptides. Alignment of the predicted recognition and core sequences shows the core peptides are 4–5 amino acids in length and appear to exclusively encode for cyclopeptide alkaloids due to the invariant C-terminal Tyr residue (Figure 3).

#### Rubiaceae family

Our previous results revealed *Coffea arabica* (Arabica coffee plant), to be a cyclopeptide alkaloid producer [7]. In contrast to those from the Rhamnaceae family, these cyclopeptide alkaloids were only cyclized and lack an oxidative decarboxylation of the C-terminus (Figure 3, arabipeptin A)*.* Our transcriptome mining results further support the prevalence of cyclopeptide alkaloids in the Rubiaceae family. *Chiococca alba* (Snowberry), *Cinchona calisaya* (Yellow Cinchona), *Gardenia jasminoides* (Cape Jasmine), *C. arabica*, and *Coffea eugenioides* (Eugenioides coffee plant) all contained >30 unique transcripts for potential precursor peptides. The core sequences themselves were typically 4 or 5 amino acids in length and most often contained a tyrosine for cyclization. However, a few sequences appeared to have a tryptophan at the position for cyclization, indicating they may correspond to the stephanotic acid-type burpitides like moroidin (Trp-indole-C to carbon crosslink, Figure 3) [4].

#### Amaranthaceae family

The Amaranthaceae family is home to the known moroidin producer, *Celosia argentea* var. *cristata* (Cockscomb and previously *Celosia cristata*) (Figure 3) [22]. Transcriptome analysis revealed that many other species in this family and the recently merged Chenopodioideae subfamily [23] have the capacity to produce burpitides as well. For example, *Alternanthera bettzickiana* (Calico Plant) was rich in 31 potential unique precursor peptide sequences. Examination of possible cores suggested that the producers closely related to *C. argentea* (*A. bettzickiana* and *Amaranthus tricolor* [edible amaranth] are likely to make bicyclic burpitides but may vary in the cycle size and identity of the cyclized residues (Figure 3 and Supporting Information File 2).

#### Malvaceae family

The Malvaceae family contains *Hibiscus syriacus* (Rose of Sharon), the only known producer of hibispeptin-type burpitides (Figure 3, hibispeptin B) [24–25]. These molecules contain a C–C linkage between the phenol derived from tyrosine and the γ-carbon of isoleucine. As expected from previous genomic analysis [7], the transcriptome of *H. syriacus* contained precursor peptides corresponding to both hibispeptin A and B (Figure 3 and Supporting Information File 2). The closely related *Hibiscus cannabinus* possessed similar transcripts, while *Hibiscus mutabilis* (Confederate rose) appeared to code for core peptides that were only four amino acids in length and not clearly hibispeptins. Outside of the *Hibiscus* genera, *Quararibea asterolepisn, Kosteletzkya pentacarpos* (Coastal Mallow)*,* and *Talipariti hamabo* all contained core peptides indicating hibispeptin-type burpitide production.

#### Icacinaceae family

In addition to these known families of producers, our transcriptomic results suggest production of burpitides from the Icacinaceae family. In particular, *Merrilliodendron megacarpum* produced 81 unique transcripts, while *Pyrenacantha malvifolia* (Monkey chair) produced more than 20. Analysis of the predicted core sequence seemed to suggest moroidin-like molecules (Figure 3), but no cyclic peptides have been isolated from any member of this family. Unfortunately, sourcing members of this small family proved challenging and we were unable to evaluate these hypotheses.

#### Iconic moroidin producers contain split precursor peptides

**Figure 4:**
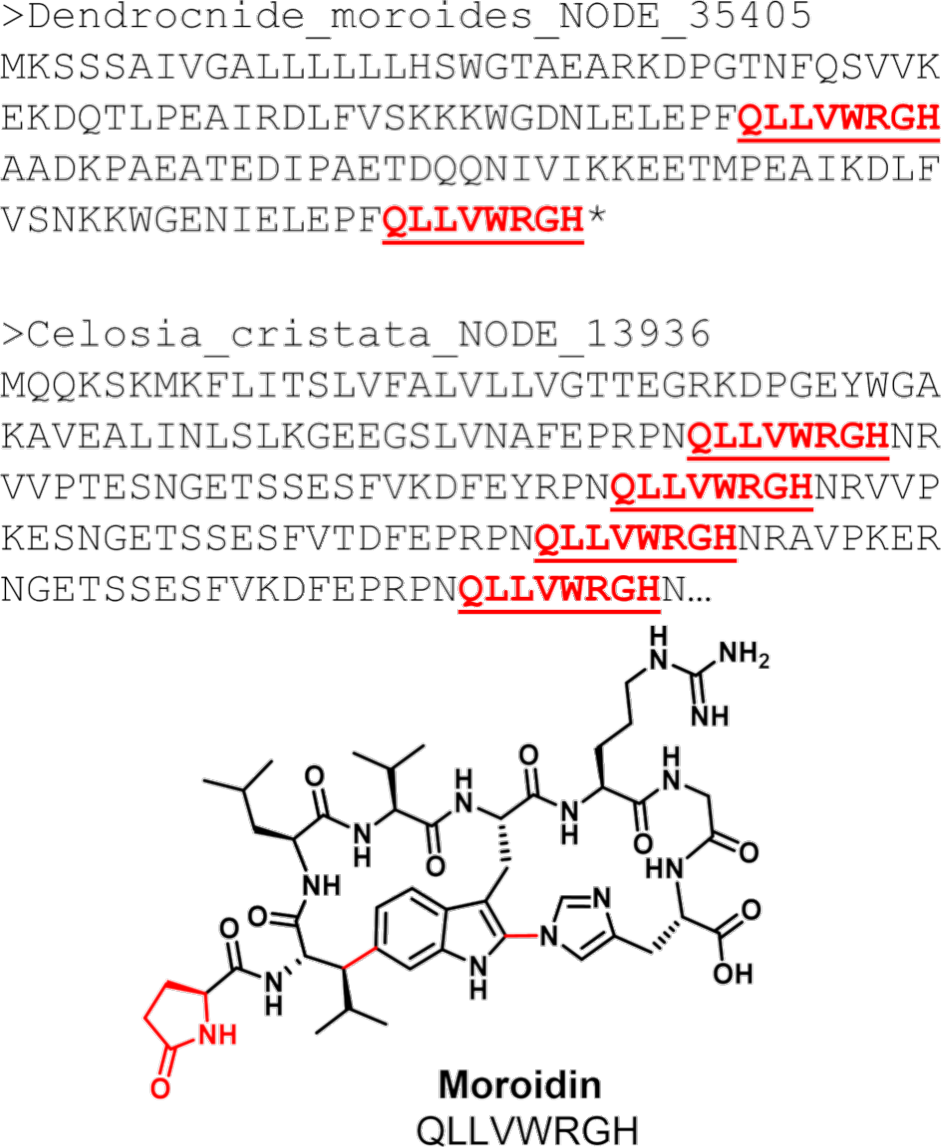
Core peptide sequences from the putative precursor peptides map to moroidin.

The moroidin family of plant peptides has previously been shown to be biosynthesized from a fused burpitide pathway using a transcriptome mining approach [9]. However, precursor peptides for the well-known producers of moroidin (*Dendrocnide moroides* [Gympie stinger] and *C. argentea*) [22,26] and the structurally related celogentin molecules (*C. argentea* var. *cristata*) [27–28] remained elusive. Using the publicly available sequencing data from *D. moroides* and *C. argentea* var. *cristana*, we could identify core sequences that perfectly matched the structure of moroidin (Figure 4). Furthermore, manually searching for the “QLLVWRGH” core sequence in the full transcriptome only revealed the stand-alone precursor peptides, further suggesting the biosynthetic pathways contain split burpitide cyclases in these plants. This seems to suggest that both split and fused burpitide biosynthetic pathways may be responsible for producing the stephanotic acid sub-family of RiPPs that is characterized by the Trp-indole-C to carbon linkage [4].

### Molecular networking of known and potential producers

**Figure 5:**
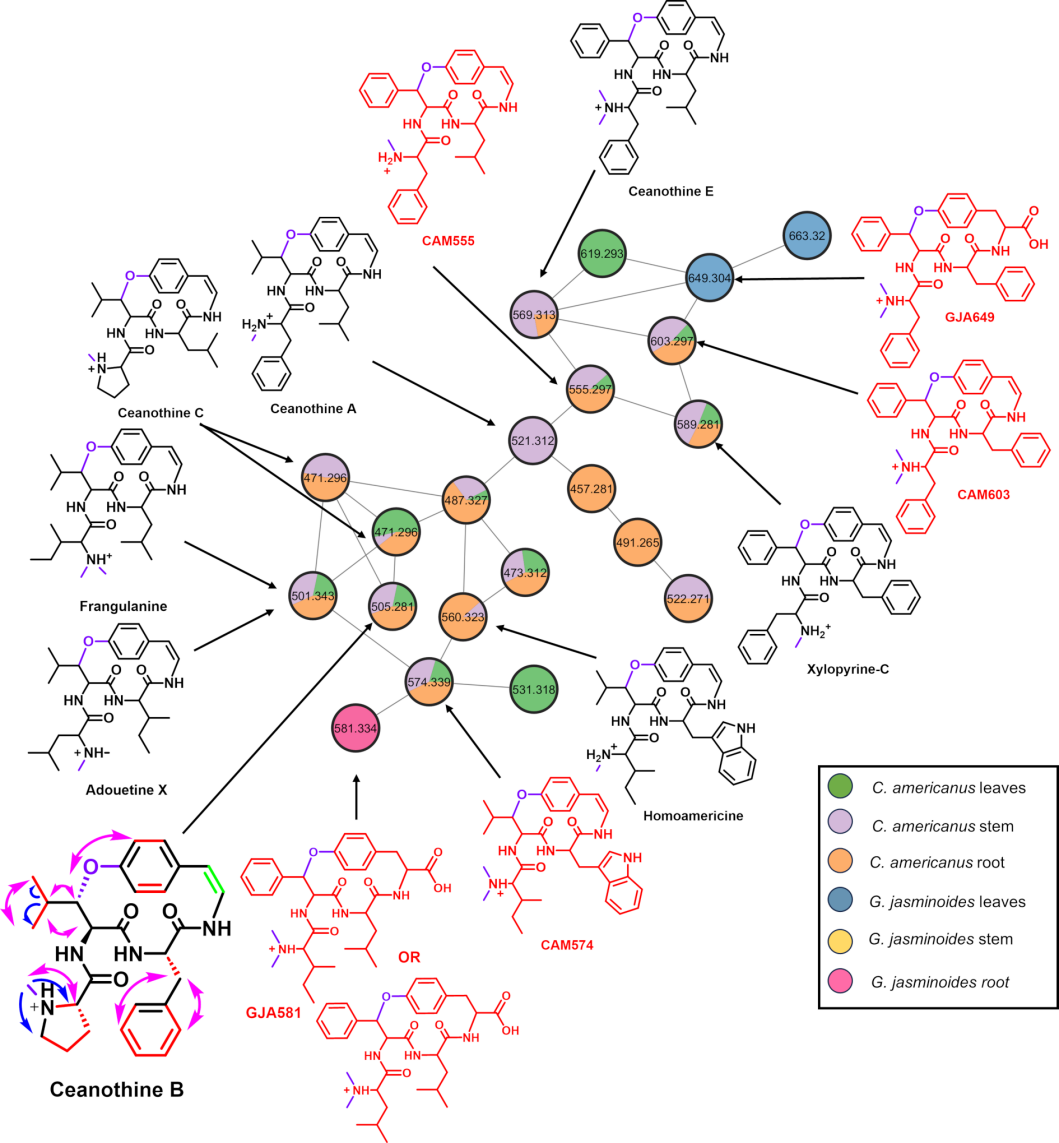
GNPS network created from extracts of *C. americanus* and *G. jasminoides*. The nodes are color-coded to show localization of various molecules. Molecules in red are new to this study and predicted by MS/MS fragmentation. Ceanothine B was isolated and its structure confirmed by 1D and 2D NMR. Correlations are indicated as follows: red lines – COSY, green lines – TOCSY, pink arrows – NOSEY, blue arrows – HMBC. Peptide modifications are shown in purple.

The transcriptome analysis indicated that many additional plant species may be responsible for the production of burpitides. To evaluate this, we examined *G. jasminoides* and *A. bettzickiana* from the Rubiaceae and Amaranthaceae families, respectively. We analyzed methanol extracts of these plants along with the known cyclopeptide alkaloid producer *C. ameranicus* using UHPLC-HRMS/MS. Finally, we generated a global natural product social (GNPS) network to show the correlation between metabolites from these different plant species (Figure 5) [29].

#### Cyclopeptides in *Ceanothus americanus*

The GNPS network showed the presence of multiple features from *C. americanus* that appeared to match known molecules such as ceanothine B (505.2809 *m/z*), ceanothine C (471.2966 *m/z*), ceanothine A (521.3122 *m/z*), ceanothine E (569.3128 *m/z*) fragulanine/adouetine X (501.3435 *m/z*), and homoamericine (560.3231 *m/z*). Despite being first isolated over 50 years ago [30–32], the majority of the proposed structures for these known cyclopeptide alkaloids are simply predicted by MS/MS fragmentation. Therefore, we isolated ceanothine B and completed full structural characterization using NMR, Marfey’s analysis, and MS/MS fragmentation (Supporting Information File 1, Figure S2, Figures S16–S26, Table S1). Indeed, our results support the proposed structure of ceanothine B while also assigning absolute stereochemistry consistent with all ʟ-amino acids (Figure 5).

We next explored the GNPS network for new cyclopeptide alkaloids from *C. americanus*. We previously noted the presence of a feature as CAM603 (603.29 *m*/*z*) [7]. Fragmentation indicated a core sequence of FFFY that was observed in precursor peptides found in the transcriptome (Supporting Information File 1, Figure S3). To further support this assignment, we partially purified the compound and completed Marfey’s analysis, which confirmed the presence of all ʟ-Phe in the structure (Supporting Information File 1, Figure S27). Beyond this, multiple other features in the GNPS network appeared to represent new molecules for *C. americanus* (Supporting Information File 1, Figures S4–S11). Xylopyrine-C was previously isolated from *Zizyphus xylopyra* [33] and matches the exact mass and fragmentation of the 589.2820 *m*/*z* feature. The 555.2953 *m*/*z* feature (CAM555) appears to be a mono-methylated version of ceanothine E. Likewise, the 574.3396 *m*/*z* (CAM574) feature is consistent with a methylated analogue of homoamericine.

#### New cyclopeptide alkaloids in *Gardenia jasminoides*

**Figure 6:**
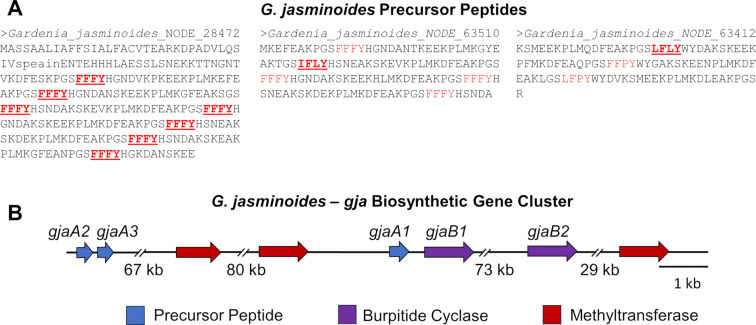
A) Precursor peptide sequences found in the transcriptome of *G. jasminoides* containing the cores FFFY, IFLY, and LFLY. A) GJA649 gene cluster from *G. jasminoides.*

The GNPS network also suggested the presence of new cyclopeptide alkaloids from *G. jasminoides*, a previously unknown producer (Figure 5). This data corroborates the transcriptome mining data where *G. jasminoides* was shown to possess an average of 50 unique precursor peptide transcripts. The 649.3047 *m/z* feature, which we named GJA649, had an exact mass and fragmentation suggested a molecule with an FFFY core (Supporting Information File 1, Figure S12). Indeed, the transcript data assembled as part of this study identified multiple transcripts from *G. jasminoides* that directly match this FFFY sequence (Figure 6A). Like other members of the Rubiaceae family [7,34], the tyrosine that forms the ether linkage remains intact, and not oxidatively decarboxylated as seen in members of the Rhamnaceae family. It is also predicted to contain a dimethylation at the N-terminal Phe residue. Additional 663.3192 and 581.3345 *m*/*z* features were also present in the GNPS network, but MS/MS fragmentation could not definitively support a specific structure.

For a better understanding of the biosynthesis of burpitides in *G. jasminoides*, we explored the genomic context by using the burpitide cyclase sequence and precursor peptides found during transcriptome assembly by performing a tblastn search against the unannotated *G. jasminoides* genome (NCBI GenBank assembly: GCA_013103745.1). Gene matches were primarily found on chromosome 4 (NCBI GenBank assembly: CM023098.1) with the location of the biosynthetic gene cluster (BGC) on the chromosome between base pairs 8,210,000 and 8,480,000. The BGC contained precursor peptides with the core FFFY (*gjaA1)*, FLFY (*gjaA2)*, and FLLY (*gjaA3),* three potential methyltransferases, and two complete burpitide cyclases all within 266,286 base pairs (Figure 6B). While the proposed gene cluster is missing a potential peptidase, it contains all the other proteins necessary for the biosynthesis of GJA649 and the other putative *G. jasminoides* cyclopeptide alkaloids.

### New cyclopeptide alkaloids in *Alternanthera bettzickiana*

**Figure 7:**
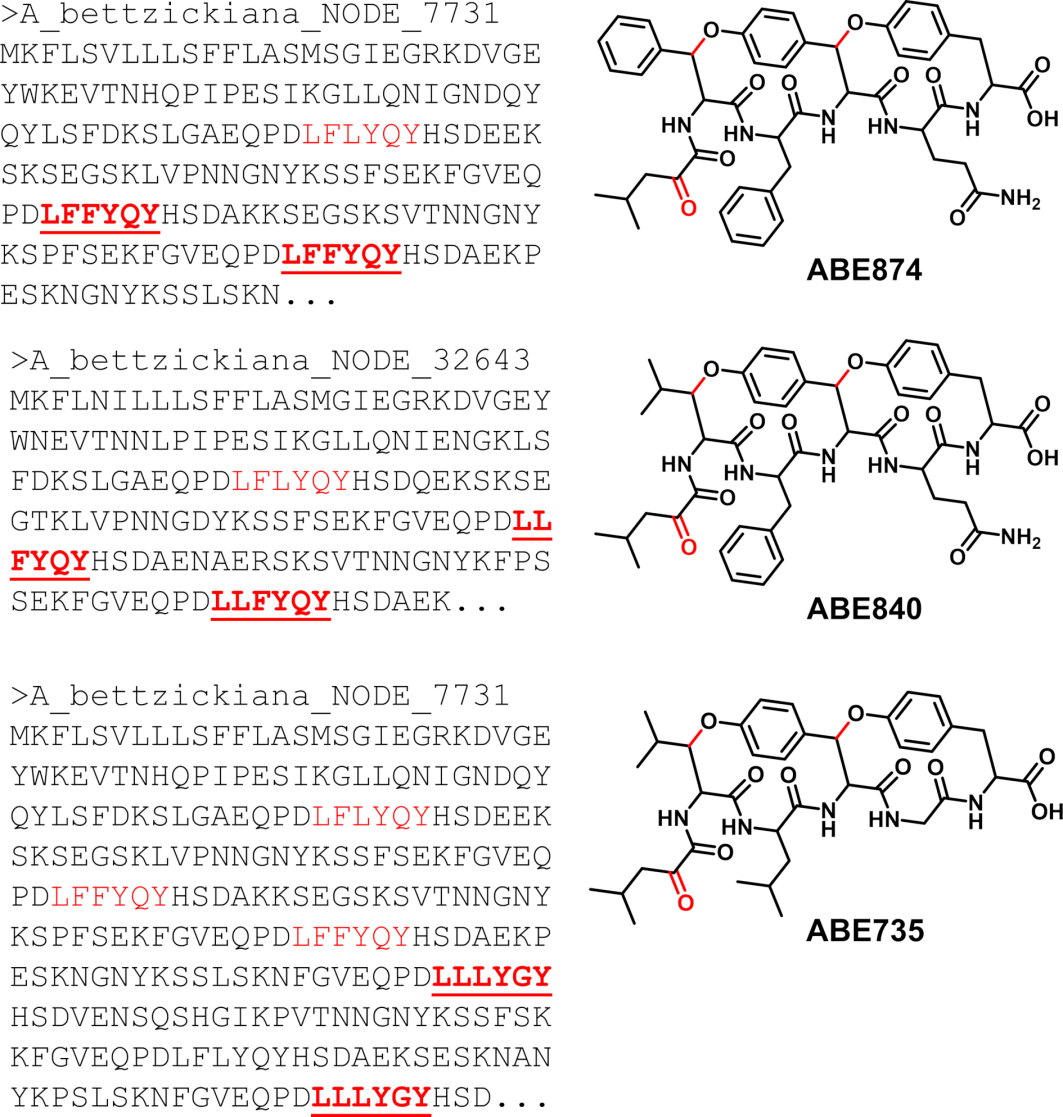
Core sequences from the putative precursor peptides map to three predicted products from *A. bettizickiana* observed by LC–MS/MS.

Based on the assembled transcriptomic data sets, it appeared that *A. bettzickiana* had the capacity to produce new cyclopeptide alkaloids. To evaluate this, a methanol extract of *A. bettzickiana* was prepared and analyzed by UHPLC-HRMS/MS. Subsequent GNPS networks did not show any clustering with cyclopeptide alkaloids from either *C. americanus* or *G. jasminoides*. Instead, the MS/MS data was searched for fragmentation patterns representing peptides, namely iminium ions [8]. Both the exact mass and fragment ions show evidence for three potential compounds with alpha-keto groups that matched putative core sequences (Figure 7 and Supporting Information File 1, Figures S13–S15). While unusual, this motif could be biosynthetically related to the alpha-hydroxy modification seen in vignatic acid [35]. These potential compounds are bicyclic which resemble the bicyclic motif found in moroidin from the closely related *C. argentea* (Figure 4). Unlike moroidins which are part of the stephanotic acid subclass of burpitides defined by the Trp-indole-C to carbon crosslink, the molecules from the *A. bettzickiana* seems to be cyclopeptide alkaloids with two tyrosine derived ether linkages that resemble selanine A isolated from African clubmoss (*Selaginella kraussiana*) [8]. To confirm these proposed structures from *A. bettzickiana*, full elucidation by NMR will be required.

### Localization of cyclopeptide alkaloids in plants

Traditionally, cyclopeptide alkaloids have been isolated from the roots and bark of the producing plants [1,19–21], likely due to the abundance of these materials. With more sensitive metabolomics techniques, we can better identify where these molecules are located in the plant. For the described GNPS network, live samples of *C. americanus* and *G. jasminoides* were processed by separating the leaves, stems, and roots. With few exceptions, cyclopeptides alkaloids from *C. americanus* were found distributed throughout multiple tissues in the plant and not only localized to the roots (Figure 5). In contrast, the cyclopeptide alkaloids in G. *jasminoides* appear to be exclusively present in either the leaves or root. As the biological functions of these molecules are currently unknown, their localization may give insights into their physiological purpose.

## Conclusion

Overall, this study illustrates the ability to rapidly assemble and analyze public transcriptomic data for the targeted discovery of new burpitide natural products from plants. Phylogenetic visualization of the potential precursor peptides validated families known to make these cyclic peptides, but also suggested new producers. Finally, metabolomic analyses supported the predicted new sources of burpitides, aiding in our understanding of the precursor rulesets for the biosynthesis of this emerging class of plant natural products.

## Experimental

### Construction of precursor peptide HMM

A custom hidden Markov model designed to target the precursor peptides from split burpitide pathways was created to favor producers of known burpitides. Sequences containing cores matching known molecules such as hibispeptins, moroidins, jubanines, and lyciumins were selected for the new model (see Supporting Information). The sequences were aligned and the hmm build function of HMMER v3.3.2 was used to generate the model [36]. The HMM is available in Supporting Information File 4 compressed archive.

### Transcriptome mining and cladogram construction

Representatives of every available order of Streptophyta were selected from the NCBI Sequence Read Archive. The full list of accessions and species is available in the Supporting Table (Supporting Information File 2). rnaSPAdes was used to assemble the raw reads using the default settings [15]. This software package was chosen primarily for its speed (≈1.5 h per transcriptome), which was essential for this large analysis effort. Our custom HMM was used to search for potential precursor peptides using an E-value inclusion threshold of 0.1. These results are present in Supporting Information File 4 compressed archive file. To remove fused burpitide cyclases, the HMM for the BURP domain (PFAM 03181) was also run against the assembled transcriptomes using the same 0.1 E-value inclusion threshold. Transcriptomes that indicated a BURP domain were removed from the putative precursor peptide list. The resulting precursor peptides were counted and mapped as bar graphs onto a cladogram of the species phylogeny. phyloT was used to generate the cladogram and R-studio and ggplot2 were used to visualize it with the number of transcripts [37–38]. For our analysis, the previously distinct Chenopodioideae family was viewed as members of the Amaranthaceae family based on the most up to date Angiosperm Phylogeny Group classification (APG IV) [23].

### Isolation of burpitides from *C. americanus*

Three separate extractions were performed overnight with methanol using 200 g of ground root powder (Mountain Rose Herb Lot# 26356) and 1.5 L of methanol. After overnight extraction, vacuum filtration was used to separate the solids from the liquids. The methanol extract was dried and saved. The solids were extracted with methanol two more times. After the extractions were finished and dried by rotary evaporation, the extract was resuspended in basic water (pH 10 using NaOH) and vacuum filtered to remove insoluble particulates. Liquid–liquid extraction was performed three times with basic water and DCM. The DCM layer was collected and dried. Afterward, the extract was resuspended in 0.1% formic acid in water.

A Combiflash EZ prep system was used to purify the burpitides from *C. americanus*. Water with 0.1% formic acid (A) and methanol (B) were used as solvents for a reversed phase column (C18 21 × 250 mm Kinetix 5 µm C18 100 Å (00G-4601-P0-AX5) with a 30–80% B gradient over 50 min at 10 mL/min. Roughly 100 mg of sample was loaded using 5 mL of a 25% MeOH/H_2_O solution. The fractions were tested using UHPLC-HRMS/MS for purity and presence of desired burptidies. Ceanothine B was successfully isolated using this approach. For CAM603, an additional purification step using an HPLC system (Agilent) with a C18 column (C18 250 × 4.6 mm Luna Omega 5 5 µm C18 100 Å) and gradient of 55–70% B was employed.

### Structure elucidation of ceanothine B

Ceanothine B was analyzed by 1D and 2D NMR in methanol-*d*_4_ in 5 mm NMR tubes (Wilmad LabGlass). NMR instrumentation was a JEOL ECA-500 MHz NMR spectrometer (JEOL Ltd.). ^1^H NMR (500 MHz, methanol-*d*_4_) δ 7.17 (m, 2H), 7.12 (m, 2H), 7.09 (m, 2H), 7.04 (m, 1H), 7.01 (m, 1H), 6.98 (m, 1H), 6.96 (m, 1H), 6.73 (d, *J* = 7.5 Hz, 1H), 6.19 (d, *J* = 7.0 Hz, 1H), 4.80 (dd, *J* = 2.0 Hz, *J* = 8.0 Hz, 1H), 4.42 (d, *J* = 8.0 Hz, 1H), 4.42 (m, 1H), 3.05 (m, 1H), 2.85 (d, *J* = 13.5 Hz, 1H), 2.74 (dd, *J* = 4.0 Hz, *J* = 10.0 Hz, 1H), 2.59 (t, *J* = 12.5 Hz, 1H), 2.33 (m, 1H), 2.16 (m, 1H), 1.97 (s, 1H), 1.95 (m, 1H), 1.79 (m, 1H), 1.68 (m, 2H), 1.15 (d, *J* = 7.0 Hz, 1H), 0.926 (d, *J* = 7.0 Hz, 1H)

Ceanothine B macrocyclization between Tyr-3 and Leu-β is supported by HMBC correlation between Tyr-C3 (δ 156.3 ppm) and Tyr-C4 (δ120.5 ppm) and NOESY correlation between Tyr4-H (δ 7.01 ppm, 1H) and Leuβ-H (δ 4.80 ppm, 1H). Similar to other characterized cyclopeptide alkaloids, *J*-coupling constant (*J*_α-β_ = 8.0 Hz) supports *S*-stereochemistry at the β-carbon of leucine ether linkage. Structurally related cyclopeptide alkaloids have similar *J*-values (*J*_α-β_ ≈ 8.0 Hz) and have been assigned as ʟ-erythro (*anti*) conformation [20,39].

### Metabolomic analysis and GNPS networking

Live samples of *C. americanus* and *G. jasminoides* were separated into leaves, stems, and roots and lyophilized for extraction. *G. jasminoides* samples were prepared by extracting 89.24 g of dried leaf material over three days with 1 L of methanol each day yielding 19.10 g of crude extract. The root and stem extracts were prepared by extracting 180.6 mg and 145.2 mg of plant material, respectively, in methanol overnight. *A. bettzickiana* was lyophilized as the whole plant. Each sample was extracted separately in methanol overnight and the extract was dried by rotary evaporation. All the residues were diluted in methanol to 2 mg/mL, except for the *G. jasminoides* leaves (1.5 mg/mL). UHPLC-HRMS/MS data was collected using an Q Exactive Plus (Thermo Scientific) coupled to a Acquity UPLC (Waters), and Acquity UPLC BEH 1.7 μm C18 reversed phase 130 Å 2.1 × 50 mm column. The following gradient was used at 1 mL/min: 0 min, 5% B; 1 min, 5% B; 11 min 100% B; 12 min, 100% B; 12.1 min, 5% B; 13 min, 5% B where solvent A was water with 0.1% of formic acid and solvent B was acetonitrile with 0.1% of formic acid. A top 5 HCD method at 25 NCE (200–2000 *m*/*z*) was used on each plant tissue extract. See Supporting Information File 1, Table S2 for complete acquisition parameters. The mass spectra were used to generate a GNPS network using the following parameters: 0.2 Da precursor ion mass tolerance, 0.5 Da fragment ion tolerance, and a minimum of 8 matched fragment ions.

### Marfey’s analysis

To determine the stereochemistry of the new molecules, Marfey’s analysis was used. Standards were prepared to match the core amino acids of the predicted ceanothine B and CAM603 cyclopeptide alkaloids. To make each amino acid standard, 0.2 mg of the ʟ- and ᴅ-amino acid was aliquoted into separate reaction vials. To each vial, 50 µL of water, 20 µL of 1 M NaHCO_3_, and 100 µL 1% Marfey’s reagent (sodium (2,4-dinitro-5-fluorophenyl)-ʟ-alaninamide) in acetone were added. The reaction mixture was incubated at 40 ºC for 1 h with periodic agitation. The reactions were quenched by adding 10 µL of 2 M HCl and dried under a nitrogen stream. Dried samples were dissolved in 1.7 mL of MeOH and injected both individually and as a combined mixture (5 µL) onto UPLC-MS (Thermo Orbitrap Elite mass spectrometer coupled to a Dionex Ultimate 3000 UPLC), with a gradient of 15–50% B over 10 min on a Kinetex 1.7 µm C18 100 Å 50 × 2.1 mm LC column using solvents of A: 0.1% formic acid in water and B: 0.1% formic acid in acetonitrile. Products were observed at 340 nm and monitored by their expected [M + H]^+^ ion.

To hydrolyze the isolated burpitides, 0.2 mg of sample was aliquoted into reaction vial. To each vial, 0.5 mL of 6 M HCl was added. The sample was incubating for 24 h at 90 ºC and dried under a nitrogen stream. Afterward, 25 µL of water, 10 µL of 1 M NaHCO_3_ and 50 µL of 1% Marfey’s reagent in acetone were added to the reaction vial. The reaction was incubated for 1 h at 40 ºC with periodic agitation. The reaction was quenched by adding 5 µL of 2 M HCl and dried under a nitrogen stream. The dried peptides were resuspended in 200 µL of MeOH. The sample was injected (6 µL) onto UPLC-MS using the same conditions and instrument as the amino acids standards. To normalize the retention time, 1 µM of fluorescein was added to the standard and peptide samples before injection onto the UPLC-MS. The retention time for the fluorescein sample was adjusted to 6.30 min to correct for run to run drifts in retention time.

## Supporting Information

The sequencing data used in this study is openly available in the NCBI SRA.

File 1 Additional details and figures including NMR spectra and MS/MS fragmentation.

File 2 Specific dataset used are listed in this Supporting Table File along with core peptide sequence alignments.

File 3 The full cladogram with species names as a high-resolution pdf.

File 4 The split burpitide precursor peptide HMM and output from all 700 transcriptomes as a compressed archive.

## Data Availability

All data that supports the findings of this study is available in the published article and/or the supporting information to this article. The data used for/in this study is openly available in NCBI SRA. Specific dataset used are listed in Supporting Table.
