# Supplementary material for: Mining raw plant transcriptomic data for new cyclopeptide alkaloids
Source: Beilstein J Org Chem. 2024 Jul 11;20:1548–59. doi: 10.3762/bjoc.20.138 (PMC11250218; doi:10.3762/bjoc.20.138)
Supplement: File 1 — Additional details and figures including NMR spectra and MS/MS fragmentation. [file Beilstein_J_Org_Chem-20-1548-s001.pdf]

BEILSTEIN JOURNAL OF ORGANIC CHEMISTRY

Supporting Information

for

Mining raw plant transcriptomic data for new cyclopeptide alkaloids

Draco Kriger, Michael A. Pasquale, Brigitte G. Ampolini and Jonathan R. Chekan

Beilstein J. Org. Chem. **2024,** *20,* 1548–1559. doi:10.3762/bjoc.20.138

Additional details and figures including NMR spectra and MS/MS fragmentation

License and Terms: This is a supporting information file under the terms of the Creative Commons Attribution License (https://creativecommons.org/licenses/by/4.0). Please note that the reuse, redistribution and reproduction in particular requires that the author(s) and source are credited and that individual graphics may be subject to special legal provisions.
The license is subject to the *Beilstein Journal of Organic Chemistry* terms and conditions: (https://www.beilstein-journals.org/bjoc/terms)

Table of Contents

1. Data Deposition

Plant Vouchers S3

NMR S3

Mass Spectra S3

2. Sequences

Sequences used for Split Burpitide HMM S4

3. Supplementary Figures and Tables

Figure S1. Visualization of HMM S8

Figure S2. MS/MS fragmentation of ceanothine B S9

Figure S3. MS/MS fragmentation of CAM603 .... S10

Figure S4. MS/MS fragmentation of xylopyrine-C S11

Figure S5. MS/MS fragmentation of CAM574 .... S12

Figure S6. MS/MS fragmentation of ceanothine E S13

Figure S7. MS/MS fragmentation of homoamericine S14

Figure S8. MS/MS fragmentation of CAM555 .... S15

Figure S9. MS/MS fragmentation of ceanothine A S16

Figure S10. MS/MS fragmentation of adouetine X/fragulanine S17

Figure S11. MS/MS fragmentation of ceanothine C S18

Figure S12. MS/MS fragmentation of GJA649 .... S19

Figure S13. MS/MS fragmentation of ABE735 .... S20

Figure S14. MS/MS fragmentation of ABE841 .... S21

Figure S15. MS/MS fragmentation of ABE875 .... S22

Figure S16. Assigned structure of ceanothine B S23

Table S1. NMR assignments for ceanothine B S24

Figure S17. ^{1}H-NMR spectrum of ceanothine B S25

Figure S18. ^{13}C-NMR spectrum of ceanothine B S26

Figure S19. ^{1}H-^{1}H-COSY NMR spectrum of ceanothine B S27

Figure S20. ^{1}H-^{13}C-HSQC NMR spectrum of ceanothine B S28

Figure S21. ^{1}H-^{13}C-HMBC NMR spectrum of ceanothine B S29

Figure S22. ^{1}H-^{1}H-NOESY NMR spectrum of ceanothine B …………………..………………….………… S30
Figure S23. ^{1}H-^{1}H-TOCSY NMR spectrum of ceanothine B ……………..……………………..………… S31
Figure S24. Marfey's analysis for ceanothine B (Proline) ……………………..……………………..…S32
Figure S25. Marfey's analysis for ceanothine B (Leucine) ……………………..…..…..……………… S33
Figure S26. Marfey's analysis for ceanothine B (Phenylalanine) ……..…………………..……………… S34
Figure S27. Marfey's analysis for CAM603 (Phenylalanine) ……………………..…………………… S35
Table S2. A table of the MS data acquisition method parameters……………………………………...S36

Data Deposition

Plants

Ceanothus americanus was purchased from Prairie Nursery (Westfield, WI 53964) and voucher deposited as NCU Accession # 677816.

Alternanthera bettzickiana was purchased from HG-USA and voucher deposited as NCU Accession # 684280

Gardenia jasminoides was purchased from Southern Living Plant Collection and voucher deposited as NCU Accession # 684279.

NMR

NMR of ceanothine B was deposited to NP-MRD as NP0051095.

Mass Spectrometry

Mass spectrometry data of ceanothine B was deposited to GNPS as MSV000094910.

Metabolomic data was deposited to GNPS as MSV000094909.

Sequences Used for Construction of Split Burpitide HMM

>XP_039019292.1 uncharacterized protein LOC120150731 isoform X8 [Hibiscus syriacus]
MKSTLVFFAFLCILLFTKTIAARKAPGEECNDETKEKFQTETTKEAKQQYVINQIPLFYYPKNVDGQEMDNAGKELAINQIPLLYYPKKDDG
QEMDKARKESVINQIPLLYYPKKVDGQEMDNAGKESDINQIPLFYYPKKVDGQEMDNAGKEFVINQIPLFYYPKKVDGQKMDKAGKESVI
NQIPLLYYPKKVDGQEMDNARKESVINQIPLLYYPKKVDGQKMDNAGKESDINQIPLFYYPKKVDGQEMDNAGKESDINQIPLFYYPKKVD
GQKMEKVGKESVINQIPLLYYPKKVDGQQMDNAIKESVINQVPLVYYPKKVGSA

>XP_039019294.1 uncharacterized protein LOC120150731 isoform X10 [Hibiscus syriacus]
MKSTLVFFAFLCILLFTKTIAARKAPGEECNDETKEKFQTETTKEAKQQYVINQIPLFYYPKNVDGQEMDNAGKELAINQIPLLYYPKKDDG
QEMDKARKESVINQIPLLYYPKKVDGQEMDNAGKESDINQIPLFYYPKKVDGQEMDNAGRESVINQIPLFYYPKKVDGQEMDNAGKEFVI
NQIPLFYYPKKVDGQKMDKAGKESVINQIPLLYYPKKVDGQEMDNARKESVINQIPLLYYPKKVDGQKMDNAGKESDINQIPLFYYPKKVD
GQKMEKVGKESVINQIPLLYYPKKVGSA

>XP_039019293.1 uncharacterized protein LOC120150731 isoform X9 [Hibiscus syriacus]
MKSTLVFFAFLCILLFTKTIAARKAPGEECNDETKEKFQTETTKEAKQQYVINQIPLFYYPKNVDGQEMDNAGKELAINQIPLLYYPKKDDG
QEMDKARKESVINQIPLLYYPKKVDGQEMDNAGKESDINQIPLFYYPKKVDGQEMDNAGRESVINQIPLFYYPKKVDGQEMDNAGKEFVI
NQIPLFYYPKKVDGQKMDKAGKESVINQIPLLYYPKKVDGQEMDNARKESVINQIPLLYYPKKVDGQKMDNAGKESDINQIPLFYYPKKVD
GQEMDNAGKESDINQIPLFYYPKKVGSA

>XP_039019287.1 uncharacterized protein LOC120150731 isoform X4 [Hibiscus syriacus]
MKSTLVFFAFLCILLFTKTIAARKAPGEECNDETKEKFQTETTKEAKQQYVINQIPLFYYPKNVDGQEMDNAGKELAINQIPLLYYPKKDDG
QEMDKARKESVINQIPLLYYPKKVDGQEMDNAGKESDINQIPLFYYPKKVDGQEMDNAGRESVINQIPLFYYPKKVDGQEMDNAGKEFVI
NQIPLFYYPKKVDGQKMDKAGKESVINQIPLLYYPKKVDGQEMDNARKESVINQIPLLYYPKKVDGQKMDNAGKESDINQIPLFYYPKKVD
GQKMEKVGKESVINQIPLLYYPKKVDGQQMDNAIKESVINQVPLVYYPKKVGSA

>XP_039019291.1 uncharacterized protein LOC120150731 isoform X7 [Hibiscus syriacus]
MKSTLVFFAFLCILLFTKTIAARKAPGEECNDETKEKFQTETTKEAKQQYVINQIPLFYYPKNVDGQEMDNAGKELAINQIPLLYYPKKDDG
QEMDKARKESVINQIPLLYYPKKVDGQEMDNAGRESVINQIPLFYYPKKVDGQEMDNAGKEFVINQIPLFYYPKKVDGQKMDKAGKESVI
NQIPLLYYPKKVDGQEMDNARKESVINQIPLLYYPKKVDGQKMDNAGKESDINQIPLFYYPKKVDGQEMDNAGKESDINQIPLFYYPKKVD
GQKMEKVGKESVINQIPLLYYPKKVDGQQMDNAIKESVINQVPLVYYPKKVGSA

>CDP09507.1 unnamed protein product [Coffea canephora]
MASSITLIAVFSIALFACITEARKNPTDFLQSAVINEHTEDNHHAESSLSNQKKTSNGNTLKDFESKPGSFLWGYQGNEAESKPKEEKPLM
KGFESKPGSFLWGYQGNDVESKSKEEKPFMKGFESKPGLLSLGVSRQQC

>XP_027065604.1 uncharacterized protein LOC113691593 isoform X3 [Coffea arabica]
MASSITLIAVFSIALFACITEARKNPTDFLQSAVINEHTEDNHHAESSLSNQKKTSNGNTLKDFESKPGSFLWGYQGNDAESKSKEEKPLM
KGFESKPGSFLWGYQGNDAESKSKEEKPLMKGFESKPGSFLWGYQGNDVESKSKEEKPLTKDFESKPGSFLWGYQGNHAESKSKKE
KPLMKDFESKPGSFLWGYQGNHAEYKEKKPLVKDN

>XP_027066141.1 uncharacterized protein LOC113692007 [Coffea arabica]
MASSITLIAVFSIALFACITEARKNPTDFLQSAVINEHTEDNHHAESSLSNQKKTSNGNTLKDFESKPGSFLWGYQGNDAESKSKEEKPLM
KGFESKPGSFLWGYQGNDAESKSKEEKPLMKGFESKPGSFLWGYQGNDVESKSKEEKPLMKDFESKPGSFL

>XP_027066439.1 uncharacterized protein LOC113692249 isoform X2 [Coffea arabica]
MASSITLIAVFSIALFACITEARKNPTDFLQSAVINEHTEDNHHAESSLSNQKKTSNGNTLKDFESKPGSFLWGYQGNEAESKSKEEKPLM
KGFESKPGSFLWGYQGNDVESKSKEEKPFMKGFESKPGSFLWGYQGNNAESKSKEENPLMKDFESKPGSFLWGYQGNDAESKSEEE
KPLMKDFELKPGSFLWGYQGNHAEYKEEKPLVKDN

>XP_027065882.1 protein PRQFV-amide-like isoform X1 [Coffea arabica]
MASSITLIAVFSIALFACITEARKNPTDFLQSAVINEHTEDNHHAESSLSNQKKTSNGNTLKDFESKPGSFLWGYQGNEAESKSKEEKPLM
KGFESKPGSFLWGYQGNDVESKSKEEKPFMKGFESKPGSFLWGYQGNNAESKSKEENPLMKDFESKPGSFLWGYQGNDAESKSEEE
KPLMKDFELKPGSFLWGYQGNDAESKSKEEKPLIKDFESKPGSFLWGYQGNDAESKSEEKKPLMKDFELKPGSFLWGYQGNDAESKS
KEEKPLIKDFESKPGSFLWGYQGNDAESKSEEKKPLMKDFELKPGSFLWGYQGNDAESKSKEEKPLIKDFESKPGSFLWGYQGPLMKD
FESKPGSFLWGYQGNDVESKSKEKKPLMKDFESKPGSFLWGYQGPLMKDFESKPGSFLWGYQGNDAEFKSKEEKPLMKDFESKPGS
FLWGYQGNHAEYKEEKPLVKDN

>XP_027065883.1 protein PRQFV-amide-like isoform X2 [Coffea arabica]
MASSITLIAVFSIALFACITEARKNPTDFLQSAVINEHTEDNHHAESSLSNQKKTSNGNTLKDFESKPGSFLWGYQGNEAESKSKEEKPLM
KGFESKPGSFLWGYQGNDVESKSKEEKPFMKGFESKPGSFLWGYQGNNAESKSKEENPLMKDFESKPGSFLWGYQGNDAESKSEEE
KPLMKDFELKPGSFLWGYQGNDAESKSKEEKPLIKDFESKPGSFLWGYQGNDAESKSEEKKPLMKDFELKPGSFLWGYQGNDAESKS
KEEKPLIKDFESKPGSFLWGYQGNDAESKSEEKKPLMKDFELKPGSFLWGYQGNDAESKSKEEKPLIKDFESKPGSFLWGYQGNDVES

KSKEKKPLMKDFESKPGSFLWGYQGPLMKDFESKPGSFLWGYQGNDAEFKSKEEKPLMKDFESKPGSFLWGYQGNHAEYKEEKPLVK
DN

>XP_027065602.1 uncharacterized protein LOC113691593 isoform X1 [Coffea arabica]
MASSITLIAVFSIALFACITEARKNPTDFLQSAVINEHTEDNHHAESSLSNQKKTSNGNTLKDFESKPGSFLWGYQGNDAESKSKEEKPLM
KGFESKPGSFLWGYQGNDAESKSKEEKPLMKGFESKPGSFLWGYQGNDVESKSKEEKPLTKDFESKPGSFLWGYQGPLMKDFESKP
GSFLWGYQGNDAESKSKEEKPLMKDFESKPGSFLWGYQGNDAESKSKEKKPLMKDFESKPSSFLWGYQGPLMKDFESKPGSFLWGY
QGNHAESKSKKEKPLMKDFESKPGSFLWGYQGNHAEYKEKKPLVKDN

>XP_027066438.1 uncharacterized protein LOC113692249 isoform X1 [Coffea arabica]
MASSITLIAVFSIALFACITEARKNPTDFLQSAVINEHTEDNHHAESSLSNQKKTSNGNTLKDFESKPGSFLWGYQGNEAESKSKEEKPLM
KGFESKPGSFLWGYQGNDVESKSKEEKPFMKGFESKPGSFLWGYQGNNAESKSKEENPLMKDFESKPGSFLWGYQGNDAESKSEEE
KPLMKDFELKPGSFLWGYQGNDAESKSKEEKPLIKDFESKPGSFLWGYQGNDVESKSKEKKPLIARLLSLGISRPSYERL

>XP_015892671.1 uncharacterized protein LOC107426888 [Ziziphus jujuba]
MKSFFALLAFSSLFLFSSTITARKEPVEYVKTGVDQVAKDLYVDPSSHILLYHGNQNGKDVAKDQSVDPSSIIPIYHGNKNGLSIDPSSIIPIY
HENRNGQSVDPLSLIPIYLGKKNGLSIDASSIIPIYHGQKNGFSVDPSSIIPIYGNHNAKDHSVDPSSLIPIYHGNQNGQSVDPSSLIPIYRDN
QNGQSVDPLSLVLLYRGNQQNGQSIDPSSLIPFYRGNQNGQFVDPSSLIPFYRGNQNGQSVDPSSLIPFYRGNQNGQSVDPSSLIPFYR
GKQQNGQFVDPSSLIPFYRGNQNGQSVDPSSFIPLYRGNQNGDQNFKANEEGVAKSVSTDQ

>XP_024932826.1 uncharacterized protein LOC107426820 [Ziziphus jujuba]
MKSFFALLAFSSLLLLSSTITARKEPGEYVKTVVEQVAEDLFVDPSSIIPFYHKNKNGQSVDPLSFLPIYHGNQNGQSIDPSSLLLIYHGNQI
GQSVDPSSFLPIYHSNQNGQSVDPSSFLPIYHGNRNGQSVDPSSFLPIYHGNRNGQSVDPSSFLPIYHGNRNGQSVDPSSFLPIYHGNQ
NGQSVDPSSFLPIYHGNRNGQSVDPSSFLPIYHGNLNRQSVDPSSFLPIYHGNRNGQSVDPSSFLPIYHGNLNGQSVDPSSFLPIYHGNR
NGQSVDPSSFLPIYHGNLNGQSVDPSSFLPLYHGNRNGQSVDPSSFLPIYHGNLNGQSIDPSSRNGQSVDPSSLLLIYRDNQNGEQNPK
ANEEGVAKSISTDQ

>XP_024932849.1 uncharacterized protein LOC107426823 [Ziziphus jujuba]
MKSFFALLAFSSLLLLSSIVIARKEPVEYVKTVVEQVAKDLSIDPSSRWPIYHGNQNGQSVDPLSRWPIYHGNQDQQFVDPLSRWPIYNG
NQNGQSVDPLSRWPIYHGNQNGQSVDPSSRWPIYHGNQNGQSVDPSSRWPIYHGNQNGQSVDPSSRWPIYHGNRNGKSVDPSSWW
PIYQNGQSVDPSSWWPIYQNGQFVDPSSRWPIYHGNQNRQSVDPSSRWPIDHGNQNGQSVDPSSRWPIYHGNQNGQSVDPSSKWPI
YHGNQNGDQNSKANEEGAAKSASTDQ

>XP_024933341.1 uncharacterized protein LOC107426824 isoform X1 [Ziziphus jujuba]
MKSFFALLAFSSLLLLSSIVIARKEPVEYVKTVVEQVAKDLSIDPSSRFPIYHDNQNGQSVDTSSRFPIYHDNQNGQSIDPSSRFLIYHGIDP
FSRFPIYHGNQNGQSIDPSSRFPIYHGNQNGQSIDPSSQFLIYRNGKSIDPSSRFPIYHGNQNGQSVDPSSRFPIYHDNQNGQSIDPSSRF
PIYHDNQNRQSIDPSSRFPIYHGNQNGQSIDPSSRFLIYHGIDPSSRFPIYHGNQNGQSIDPSSRFPIYHGNQNGDQNSKANEEGAAKSA
STDQ

>XP_024933342.1 insoluble matrix shell protein 4-like isoform X2 [Ziziphus jujuba]
MKSFFALLAFSSLLLLSSIVIARKEPVEYVKTVVEQVAKDLSIDPSSRFPIYHDNQNGQSVDTSSRFPIYHDNQNGQSIDPSSRFPIYHGNQ
NGQSIDPSSQFLIYRNGKSIDPSSRFPIYHGNQNGQSVDPSSRFPIYHDNQNGQSIDPSSRFPIYHDNQNRQSIDPSSRFPIYHGNQNGQ
SIDPSSRFLIYHGIDPSSRFPIYHGNQNGQSIDPSSRFPIYHGNQNGQSIDPSSRFPIYHGNQNGDQNSKANEEGAAKSASTDQ

>KAH7517848.1 hypothetical protein FEM48_Zijuj09G0107700 [Ziziphus jujuba var. spinosa]
MKSFFALLAFSSLFLFSSTITARKEPVEYVKTVVDQVAKDLYVDPSSHILLYHGKQNGKDAAKDQSVDPSSIIPIYHGNKNGLSIDPSSIIPIY
HENRNGQSVDPLSFIPIYLGKKNGLSIDASSIIPIYHGQKNGFSVDPSSIIPIYGNHNAKDHSVDPSSLIPIYHGNQNGQSVDPSSLIPIYRDN
QNGQSVDPLSLVLLYRGNQQNGQSIDPSSLIPFYRGNQNGQSVDPLSLIPFYRGNQNGQSVDPSSLIPFYRGNQNGQSVDPSSLIPFYR
GKQQNGQFVDPSSLIPFYRGNQNGQSVDPSSLIPIYRGNQNGDQNFKANEEGVAKSVSTDQ

>KAH7517857.1 hypothetical protein FEM48_Zijuj09G0108600 [Ziziphus jujuba var. spinosa]
MKSFFALLAFSSLLLLSSTITARKEPGEYVKTVVEQVAEDLFVDPSSIIPFYHKNKNGQSVDPLSFLPIYHGNQNGQSIDPSSLLLIYHGNQI
GQSVDPSSFLPIYHSNQNGQSVDPSSFLPIYHGNRNGQSVDPSSFLPIYHGNRNGQSVDPSSFLPIYHGNRNGQSVDPSSFLPIYHGNR
NGQSVDPSSFLPIYHGNQNGQSVDPSSFLPIYQGNRNGQSVDPSSFLPIYQGNRNGQSVDPSSFLPIYHGNLNRQSVDPSSFLPIYHGN
LNGQSVDPSSFLPIYHGNQNGQSVDPSSFHLLYHGNRNGQSVDPSSFLPIYHGNLNGQSIDPSSRNGQSVDPSSLLLIYRDNQNGEQNP
KANEEGVAKSISTDQ

>KAH7517853.1 hypothetical protein FEM48_Zijuj09G0108200 [Ziziphus jujuba var. spinosa]
MSINTESYTQPLHHSATTHLRTLPKKERIIASHFELFQIQLVIKMKSFFALLAFSSLLLLSSTITARKEP
AEYVKTVVEQVVKDLSVHPSSAVPFYRSNKNGQSVDPSSAVPFYHENKNDLSVDPSSAVPFYRGNQNDQSVDPSSAVPFYHGNKNGL
SVDPSSAVPFYRGNQNGQFVDPSSAVPFYHGNKNSLSVDPSSAVPFYRSNQNGQSIDPSSAVPFYHRNKNGLSVDPSSALPFYRGNQ
NGQSVDPSSAVPFYHGNKNGLSVDPSSVLPFYRGNQNGQSVDPSSAVPFYHGNKNGLSVDPSSVLPFYRGNQNGQSVDPSSAVPFYH
GNKNGLSVDPSSVLPFYRGNQNGQSVDPSSAVPFYHGNKNGLSVDPSSAVPFYRGNQNGQSVDPSSAVPFYHGNKNGLSVDPSSAVP
FYRGNQNGQSVDPSSAVPFYHGNKNGFFVDPSSAVPFYHGNQNGQSVDPSSAVPFYHGNKNGFFVDPSSAVPFYHGNQNGQSVDPS
SAVPFYHGNKNGFFVDPSSAIPFYYGNKNGLYIDPSSAVPFYHSNQNNQSVDPSSAVPFYRGNQNGDQNSKANEEGATKNLPLTNEM

>KAH7517850.1 hypothetical protein FEM48_Zijuj09G0107900 [Ziziphus jujuba var. spinosa]

MNLLDIILEHKGVGEKLARSCSGVVLVPPFALSSTIIARKEPVEYVKTVVEQVAKDLSVDPSFAVPFYRSNKNGQSVDPSSAVPFHHGNKN
GLSIDPSSAIPVYRGNQNGQSIDPSAAIPIYHGNQNGQSIDPSSALLIYRINQNGLSIDPSSAVPFYHGNKNGFSIDPSSAVPFYHGNKNGL
SVDPSSAIPVYQGNQNGQSIDPSSAVPVYRSNQNGQSIDPSSAVPIYHGNKNGLSVDPSSAIFVYRGNQNGQSIDPSSAIPVYRGNQNG
QSIDPSSAIPVYRGNQNGQSIDPSSALLIYRINQNGLSIDPSSAVPFYHGKKNGFFVDPSSAVPFYHGNKNGLSVDPSSAIPVYQGNQNG
QSIDPSSAVPVYRSNQNGQSIDPSSAVPIYHGNKNGLSVDPSSAIFVYRGNQNGQSIDPSSAIPIYHGNQNGQSIDPSSAVPVYRGNQNG
QSIDPSSALLIYRINQNGLSIDPSSAVPFYHGNQNGLSMDLSSAVPFYRGIKNGLSVDPSSAVPFYYENKNGLSVDPSSAVPFYRGNQNG
EQNSKANEKGATKNLSTDQ

>XP_028789885.1 feeding circuit activating peptides-like [Prosopis alba]
MEFLRFSVLAVLFVTLIASDATSSRSAEEYWHSIWPHTPMPKNLEYLLGSGVNDAENRETQYPYTSFFEDDLHDGKEMNIIFNSPYAKEEL
KSIAMEKDAKKPPSSQPWGVGIWKRNAMNEDSKKSSSVSLQPWGVGIWKRNAMNEDSEKSFVSPQPWGVGIWKRNAMNEDSEKSLV
STQPWGVGIWKRNTMNEDSEKSLVSPQPWGVGIWKKNAMDEDSEKSSVSPQSWGVGTWKRNSMNEDSEKSSISPQPWGVGMWKIN
SMNEDSEKSSVSPQPWGVGMWKRNSMNEDSEKSSVSPQSWGVGIWKRNSMNEDSKKSSVSPQPKGVGIWKRNAMNEDIEKSTASP

>XP_028797721.1 uncharacterized protein LOC114753225 [Prosopis alba]
MGLLRFGVLALLLCFALTGSGATSSRSAEEYWHSIWPDTPLPKNLEYLLESGVTDAKNKDTQYPYTSFFEDDLHPGKQMNMIFNSPYAN
DAMHKDANKASSSQPWGVGSWKRNMMNEDLEKSSVSQPWGFGSWKRNIMNEDSEKSSVSQPWGVGSWQRNTNEESEKSSLFSQP
WGVGSWQRNTNENSEKSSYLLNPGVLIHGKET

>a feeding circuit activating peptides-like [Prosopis alba]
MEFLRFSVLAVLFVTLIASDATSSRSAEEYWHSIWPHTPMPKNLEYLLGSGVNDAENRETQYPYTSFFEDDLHDGKEMNIIFNSPYAKEEL
KSIAMEKDAKKPPSSQPWGVGIWKRNAMNEDSKKSSLVSLQPWGVGIWKRNTMNEDSEKSFVSPQPWGVGIWKRNAMNEDSEKSLV
STQPWGVGIWKRNTMNEDSEKSLVSPQPWGVGIWKKNAMDEDLEKSSVSPQPWGVGIWKRNSMNEDSEKSSVSPQPWGVGMWKIN
SMNEDSKKSSVSPQPWGVGMWKRNSMNEDSEKSSVSPQPWGVGIWKRNSMNEDSEKSSVSPQPKGVGIWKRNAMNEDIEKSTTSP

>XP_039013237.1 uncharacterized protein LOC120142811 [Hibiscus syriacus]
MFSQSAETIAARKAPDTKWTVKKEDQPTEEALKPTEHESFVKDFDPNRPQFPLIYYSNKDSKPRQPVQNDKSFVKDFDPNRPQFPLIYYS
KKDSQSEEPIETDKSFVKDFDPNRPQFPLIYYSKKDDQSQVSTENDKSFVKNFDPNRHQIPLIYYANKDGQSQEHVENDKSFVKDFDPNR
PQFPLIYYSKKDDQSQESAKTDKSFVEDSKSMLSEVEKAFVKVVNI

>XP_039017147.1 uncharacterized protein LOC120148011 [Hibiscus syriacus]
MLQPTYPSKVIGITNSEDYLAFSAIMRSFFSFFALFCVLLSAETIAARKAPDTQWTNEKEEQSMVEAVEPTERESFVKDFDPNRPQFPLVY
YSKKDGQSQEQAENDKSFIKDFNPNRPQFPLVYYSKKDGQSKEHAENDKSFVKDFDPNRPQFPLVYYSKKDGQSQEHEENDKSFVKDF
DPNRPQFPLVYYSKKDGQSQEHAKNDKSFVKDFDPNRPQFPLVYYSKKDGESREHAENDKSFVKDFDPNRPQFPLVYYSKKDGQSQE
PTKTDKVVNI

>KAF5730835.1 Cdc15p [Tripterygium wilfordii]
MRSLFAFVALLLLFLVLGSANAARTEPGGYWKAVMKDQPMPDTIRARIVSSKSNDHLQVSTENSDCDQKAEKNGVDLSGRDFDFDTKIK
PSQLLVWREHEKVDKPGMDLSSKDFDFDTEIKPSQLLVWREHKKVDKPSLDLSGKDFDFDAEIKPSQLLVWREHMKVDKSSLDLSGKDF
DFDAEIKPSQLLVWREHLDMKAKENI

>XP_038686271.1 uncharacterized protein LOC119985889 isoform X2 [Tripterygium wilfordii]
MRSLFAFVALLLLFLVLGSANAARTEPGGYWKAVMKDQPMPDTIRARIVSSKSNDHLQVSTENSDCDQKAEKNGVDLSGRDFDFDTKIK
PSQLLVWREHEKVDKPGMDLSSKDFDFDTEIKPSQLLVWREHKKVDKPSLDLSGKDFDFDAEIKPSQLLVWREHKKVDKPSLDLSGKDF
DFDAEIKPSQLLVWREHEDVKAKENI

>XP_038686270.1 uncharacterized protein LOC119985889 isoform X1 [Tripterygium wilfordii]
MRSLFAFVALLLLFLVLGSANAARTEPGGYWKAVMKDQPMPDTIRARIVSSKSNDHLQVSTENSDCDQKAEKNGVDLSGRDFDFDTKIK
PSQLLVWREHEKVDKPGMDLSSKDFDFDTEIKPSQLLVWREHKKVDKPSLDLSGKDFDFDAEIKPSQLLVWREHMKVDKSSLDLSGKDF
DFDAEIKPSQLLVWREHKKVDKPSLDLSGKDFDFDAEIKPSQLLVWREHKKVDKPSLDLSGKDFDFDAEIKPSQLLVWREHEDVKAKENI

>XP_021713887.1 uncharacterized protein LOC110681997 [Chenopodium quinoa]
MQVAWLGNIHNSCWRHLTEMKLSITSTLLLALLLVVNVAHGCNDDGGVGEYWGKKAIETLSGIKSNEERQNSGEYWGKKAIEALVGLKS
NDVHKFNSIVKDFKFKPNQWQYLDTNPKLENGETTTYFVKDFVPKPNHVLQWHHGAASHDDDEVAPIYFVKDFVPKPNHVLQWHHGAA
SHDDEVSTAKYFVRDFEPKINHVLQWHHGAANPTKDVWPTIKSVVTGFKFN

>XP_021728296.1 uncharacterized protein LOC110695359 [Chenopodium quinoa]
MKLSITSTLLLALLLVVNVAHGCNDDSVGEYWGKKAIETLSGIKSNEGPQYSGEYWGKKAIEALVGFKSNDVQKFNSIVKDFKFKPNQWQ
YLDTNPNSKNGETTSAYFVKDFVPKPNHVLQWHHGAASHDDEVSTTKYFVRDFEPKVNHVLQWHHGAANPTKDVWPTIKSVVTGFEFN

>QIG55796.1 moroidin precursor [Celosia argentea]
MKFLITSLVFALVLLVGTTEGRKDPGEYWGAKAVEALVDLSLKGEEGSLVNAFEPRPNQLLVWRGHNRVVPTESNGETSSESFVKDFEY
RPNQLLVWRGHNRVVPKESNGETSSESFLTDFEPRPNQLLVWRGHNRDVPKESNGETSSESFVKDFEPRPNQLLVWRGHNRVVSKES
NGEISSESFVKDFEPIPNQLLVWRGHNKVVPKESNGETLSESFLKDFEPRPNQLLVWRGHRKANPTENDGATTNAPFVKDLEPTSN

>QIG55798.1 moroidin precursor [Amaranthus cruentus]

MKFSLISLAFTLLLFINISEGRKDPGDYWGKKSIEALIDSNLSEDEKFIVINNFEPRPNQLLIWPRHDKVSPTETKEASVYNKHVLNNFEPRP
NQLLVWPRHNKVVPSNTNEASTSSESLVNNFEPRPNQLLVWPRHNKVSPTENKEVSISNKQVVNNFEPRPNQLLVWPRHNKVAPSDTN
EVSTSSESVVNNFEPRPNQLLIWPRHNKVAPTETSEASVSNEFVVNNFEPRPNQLLVWPRHNKVVSSDTNEASTSSESIVNNFEPRPNQ
LLVWRNHNKVSPNEINDVTISSESLAINFEPRPNQLLVWRNHNKVVPKETDEGISF

>QIG55797.1 moroidin precursor [Dendrocnide moroides]
MKSSSAIVGALLLLLLHSWGTAEARKDPGTNFQSVVKEKDQTLPEAIRDLFVSKKKWGDNLELEPFQLLVWRGHAADKPAEATEDIPAET
DQQNIVIKKEETMPEAIKDLFVSNKKWGENIELEPFQLLVWRGH

>Ceanothus_americanus_27101
MKTFFALFAFSSLLLLSSTITARKEPEYLKTVIENQPILEVLQGVLDLITKSGNGGRVAKDISVDPSGNILWYHGNKNAEAKSKDDGEHKDD
FSVDPSGNELWYHDNKNAEAKSKADGEHKDDFSVDPSGNELWYHDNKNAEAKSINPSGNVLWYHGNKNAEAKSINPSGNVLWYHGNK
NAEAKSKADGEHKDDFSVDPSGNVLWYHGKKIVELDSETIAEQVAKAFSDAPSGTFILYHGNKNGESNSKASGEGAAKDSFVDPSVFDK

>Ceanothus_americanus_27108
MKTFFVLLAFSSLLLSRTITARKEPAEYAKTIMENQPIPVLEVIQGVLDLIAKSSRNGGRLAKDLSVDPLSNWLIYRGNQNGENVAKDLKDL
SVDPSSNFLIYRGNHNGENAAKDLSVDPLSNWLIYRGNQNGENVAKDLKDLSVDPSSNFLIYRGNHNEENVAKDLSVDPSSNFLIYRGNH
NEENVAKDLSVDPSSNFLIYRGNHNEENVAKDLSVDPSSNFLIYHGNKNADPNSKANGEGVAKGISPDHHDQ

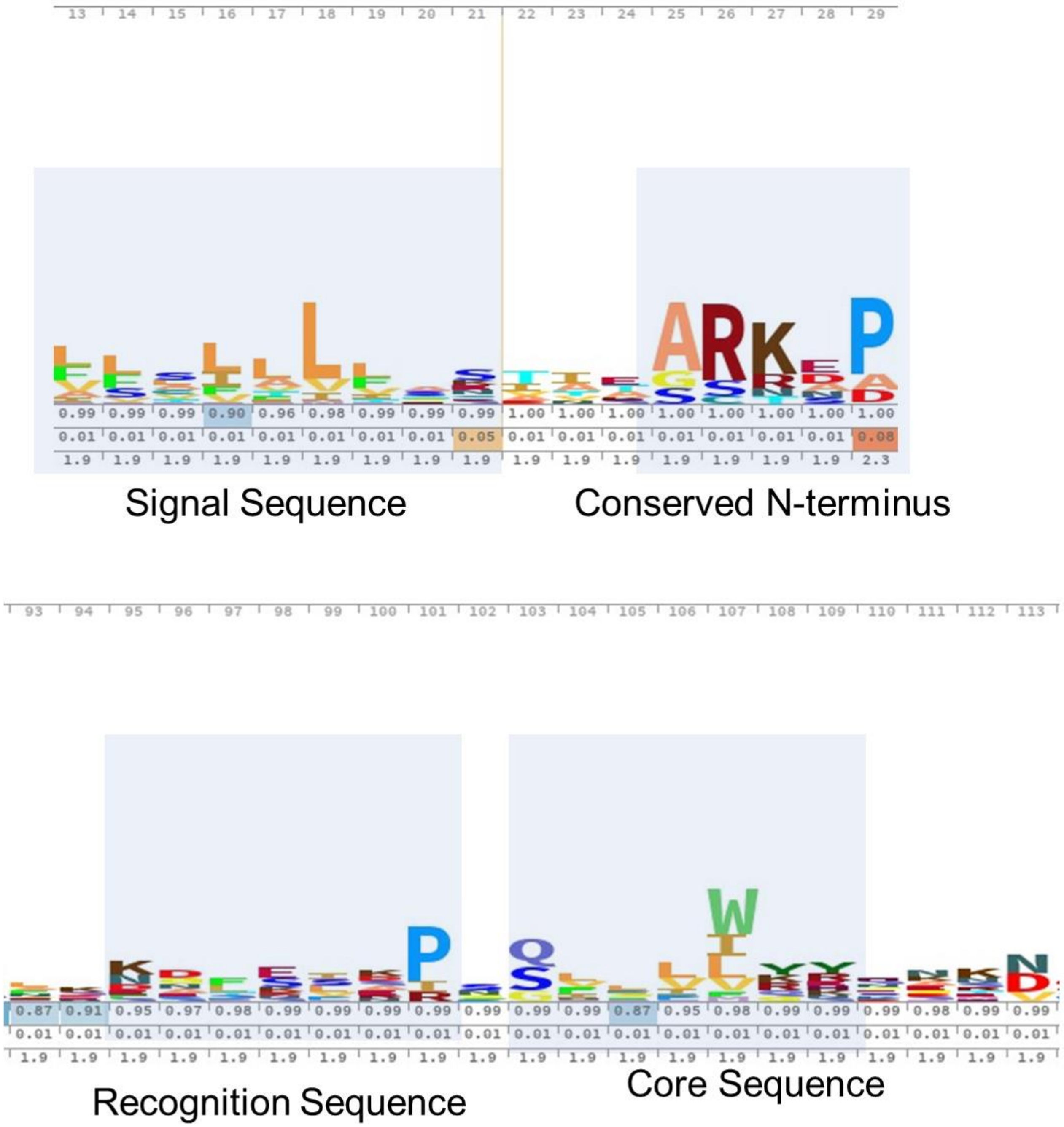

Figure S1. Visualization of Hidden Markov Model generate using Skylign (https://skylign.org/)

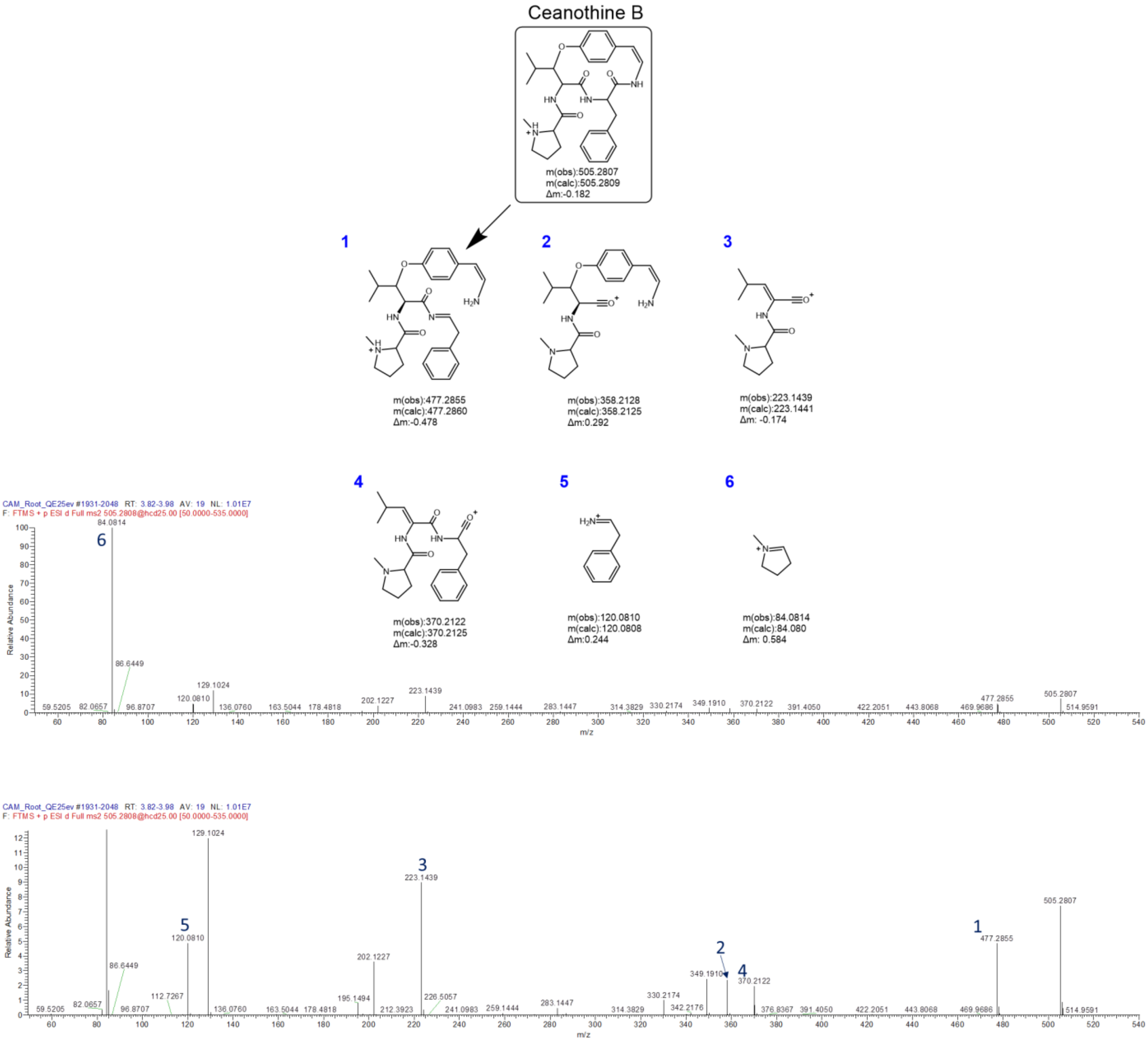

Figure S2. MS/MS spectrum of ceanothine B from *C. americanus* extract. The bottom spectrum is a zoom-in of the top spectrum. The mass error is in ppm.

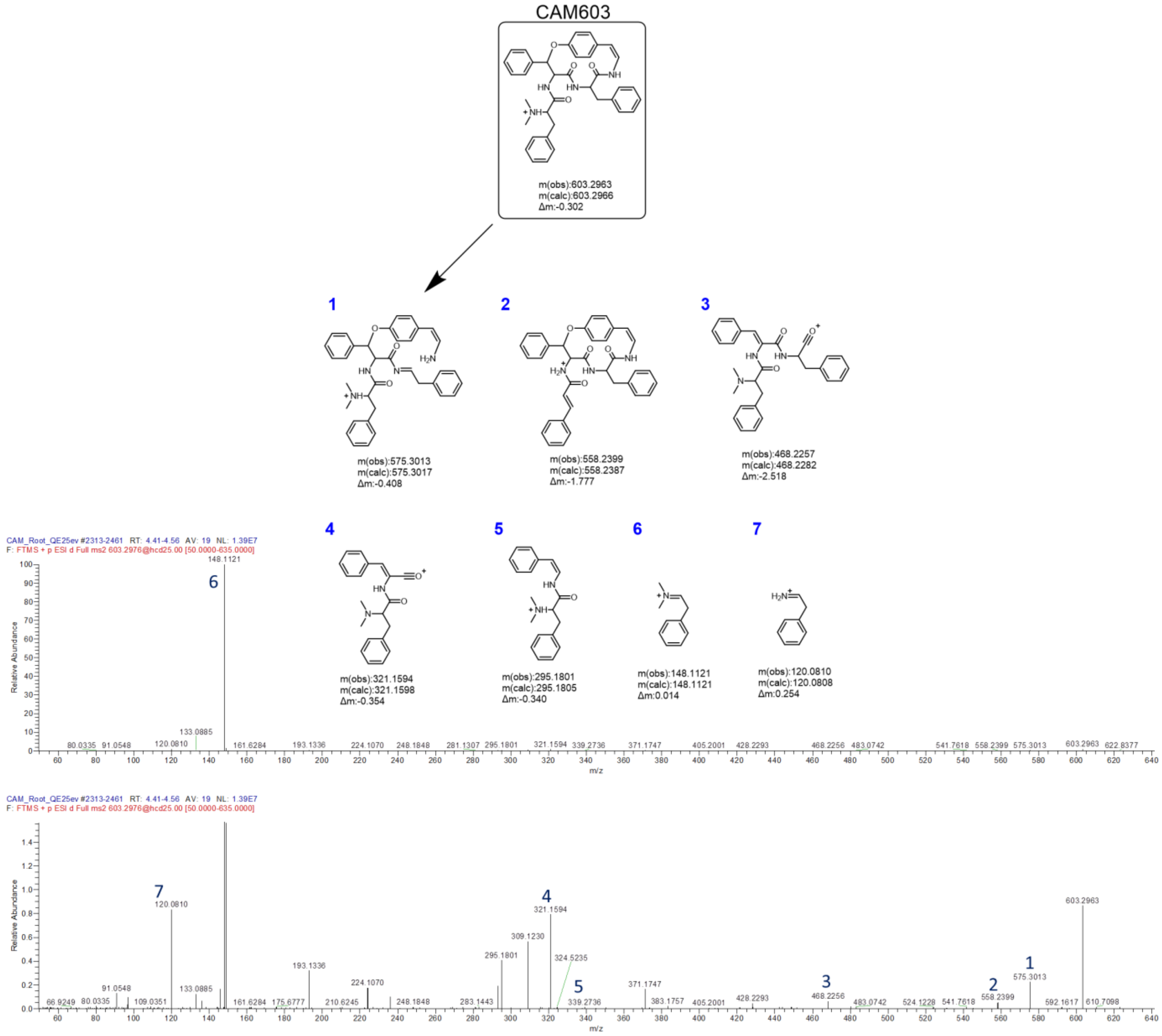

Figure S3. MS/MS spectrum of a feature from *C. americanus* extract consistent with a cyclopeptide alkaloid derived from a F-F-F-Y core. The bottom spectrum is a zoom-in of the top spectrum. The mass error is in ppm.

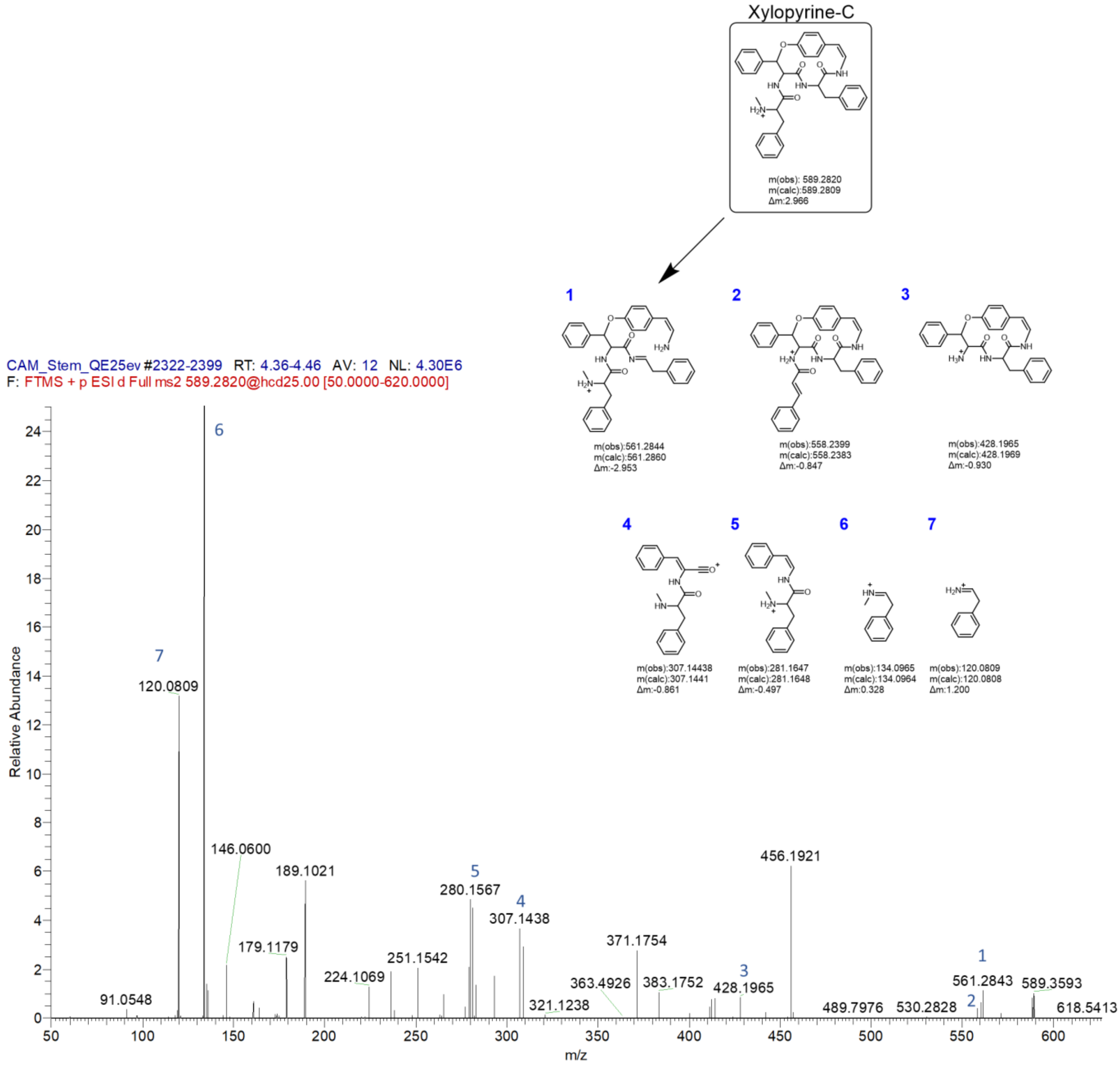

Figure S4. MS/MS spectrum of a feature from *C. americanus* extract consistent with a cyclopeptide alkaloid derived from a F-F-F-Y core. The mass error is in ppm.

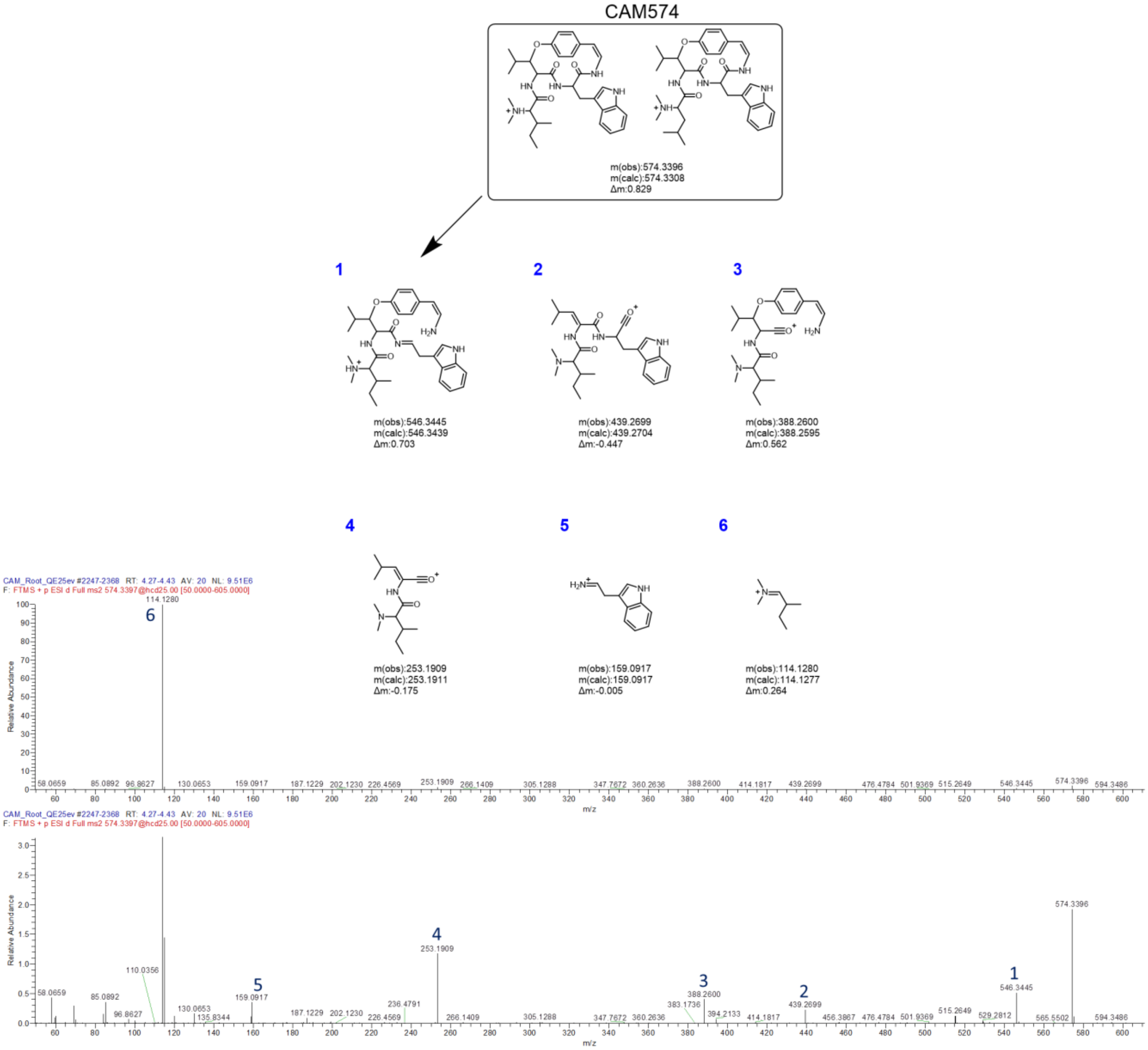

Figure S5. MS/MS spectrum of a feature from *C. americanus* extract consistent with a cyclopeptide alkaloid derived from a I/L-L-W-Y core. The bottom spectrum is a zoom-in of the top spectrum. The mass error is in ppm.

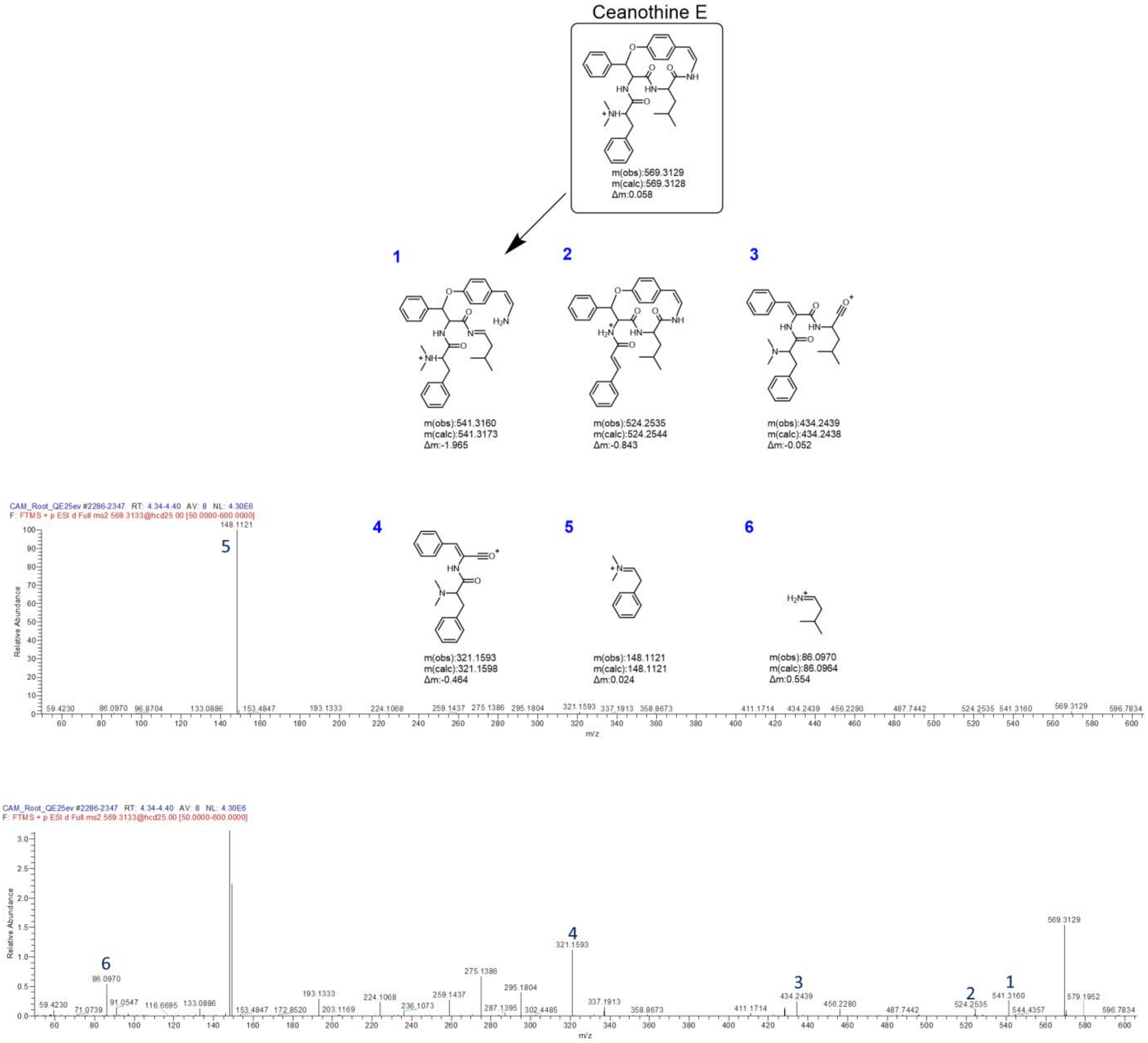

Figure S6. MS/MS spectrum of a feature from *C. americanus* extract consistent with a cyclopeptide alkaloid derived from a F-F-L-Y core. The bottom spectrum is a zoom-in of the top spectrum. The mass error is in ppm.

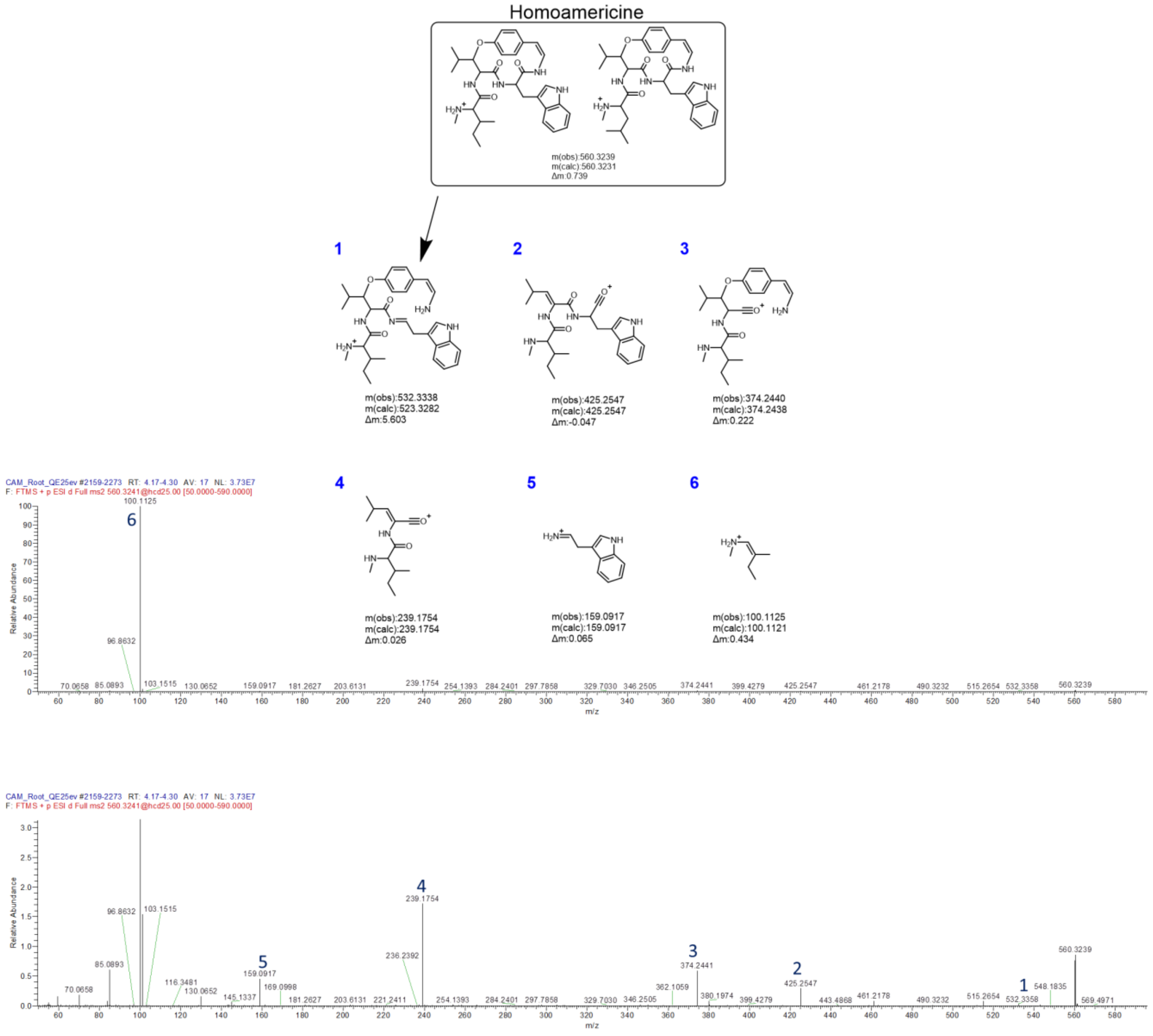

Figure S7. MS/MS spectrum of a feature from *C. americanus* extract consistent with a cyclopeptide alkaloid derived from a L/I-L-W-Y core. The bottom spectrum is a zoom-in of the top spectrum. The mass error is in ppm.

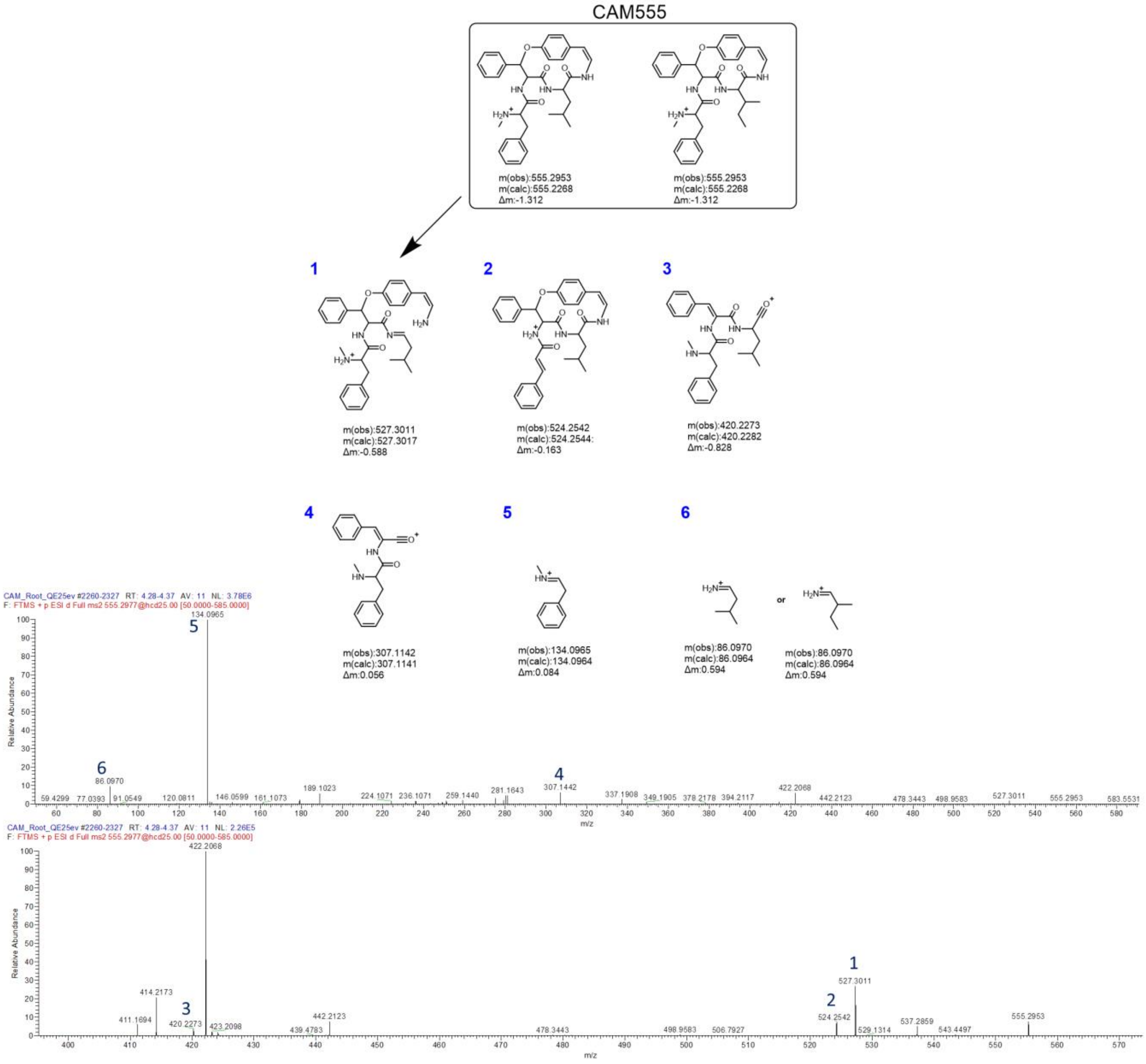

Figure S8. MS/MS spectrum of a feature from *C. americanus* extract consistent with a cyclopeptide alkaloid derived from a F-F-L/I-Y core. The bottom spectrum is a zoom-in of the top spectrum. The mass error is in ppm.

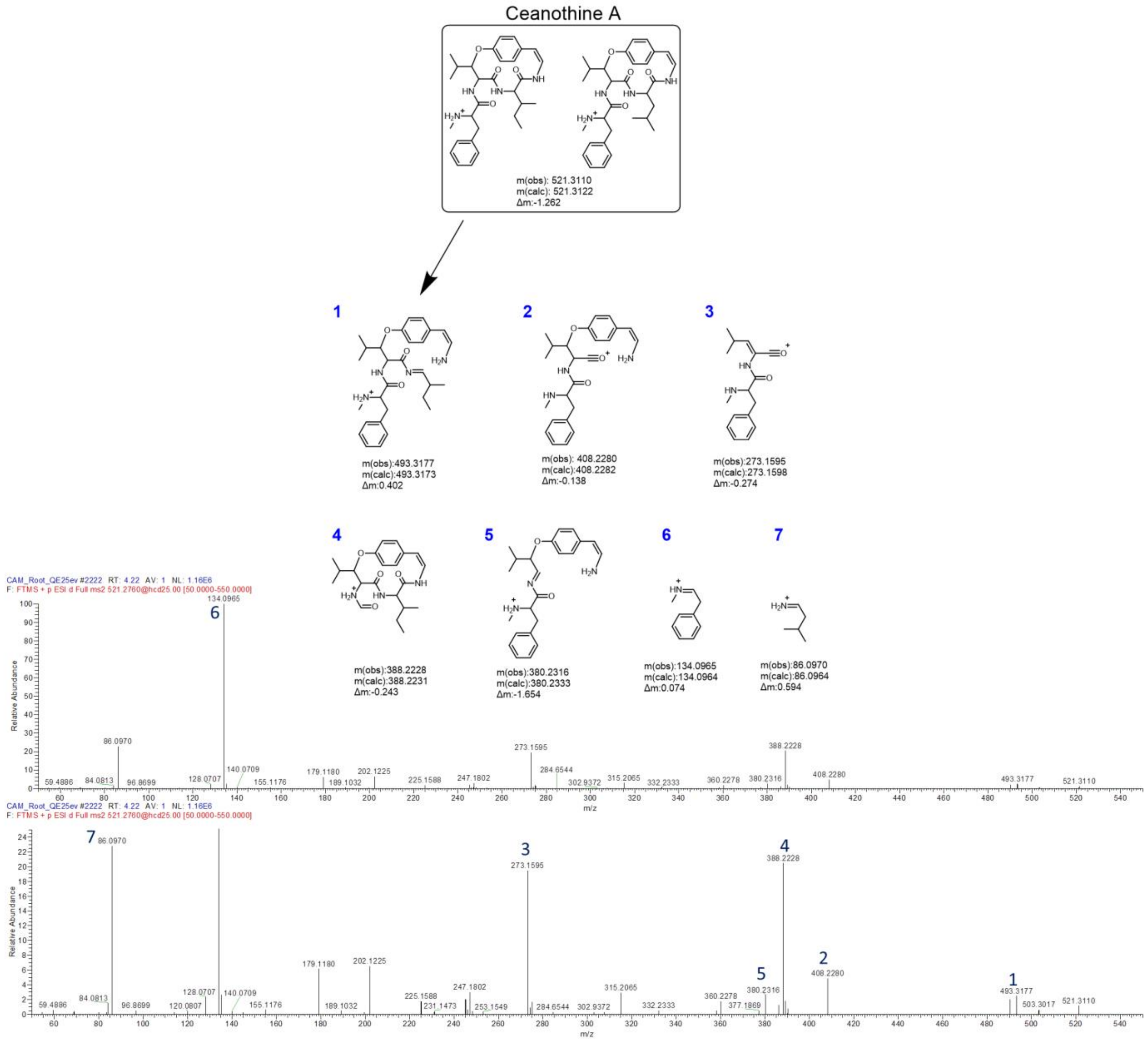

Figure S9. MS/MS spectrum of a feature from *C. americanus* extract consistent with a cyclopeptide alkaloid derived from a F-L-I/L-Y core. The bottom spectrum is a zoom-in of the top spectrum. The mass error is in ppm.

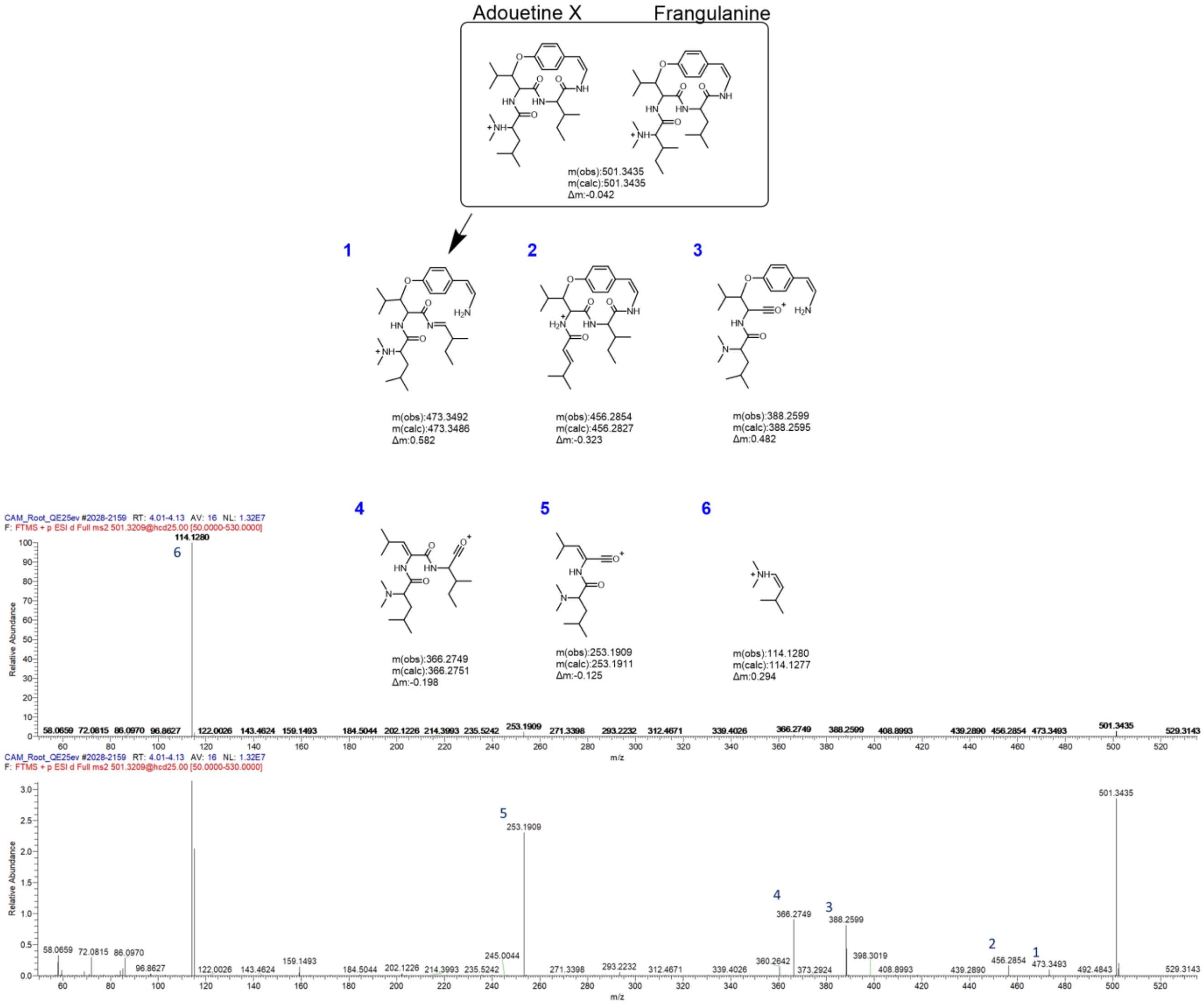

Figure S10. MS/MS spectrum of a feature from *C. americanus* extract consistent with a cyclopeptide alkaloid derived from a I/L-L-I/L-Y core. The bottom spectrum is a zoom-in of the top spectrum. The mass error is in ppm.

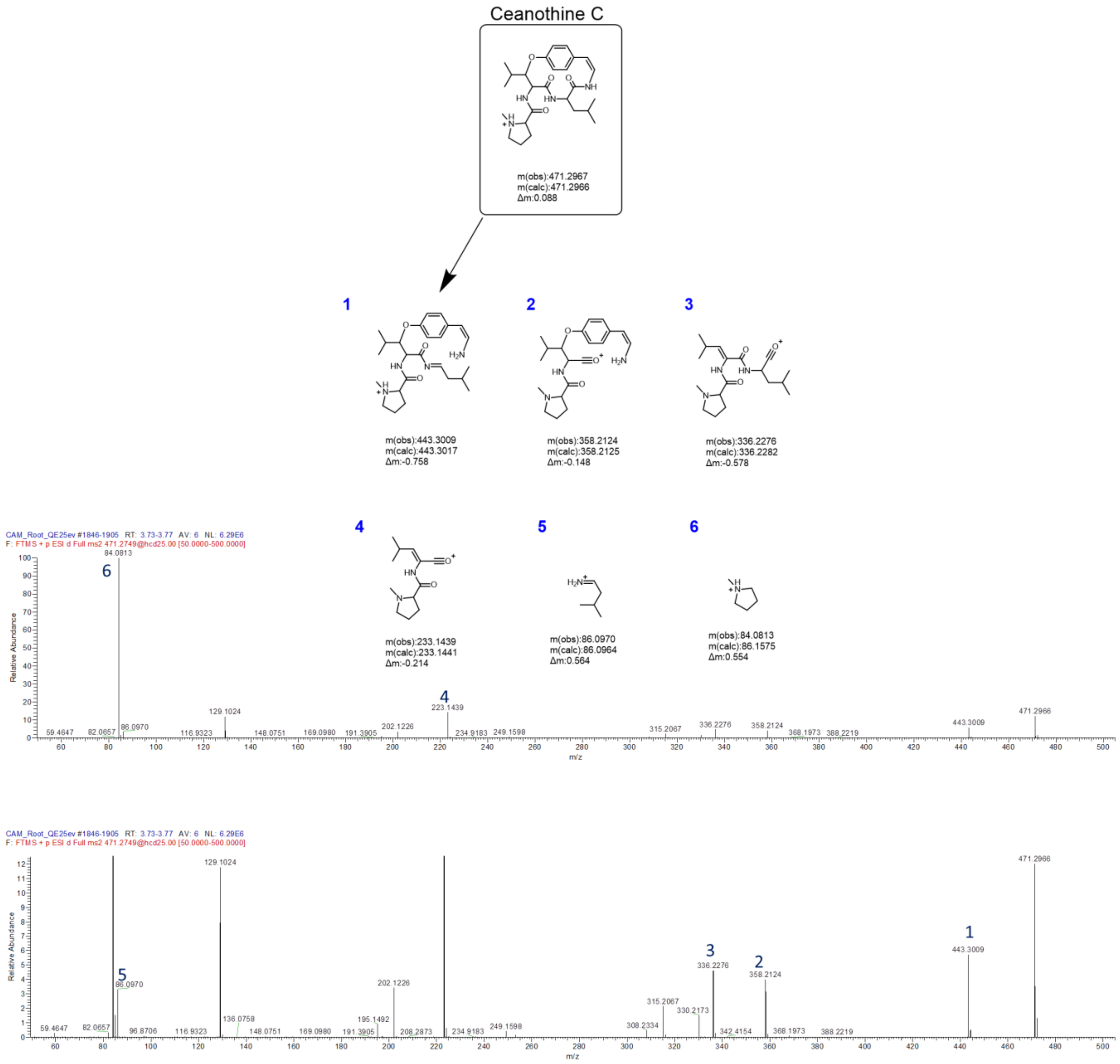

Figure S11. MS/MS spectrum of a feature from *C. americanus* extract consistent with a cyclopeptide alkaloid derived from a P-L-L-Y core. The bottom spectrum is a zoom-in of the top spectrum. The mass error is in ppm.

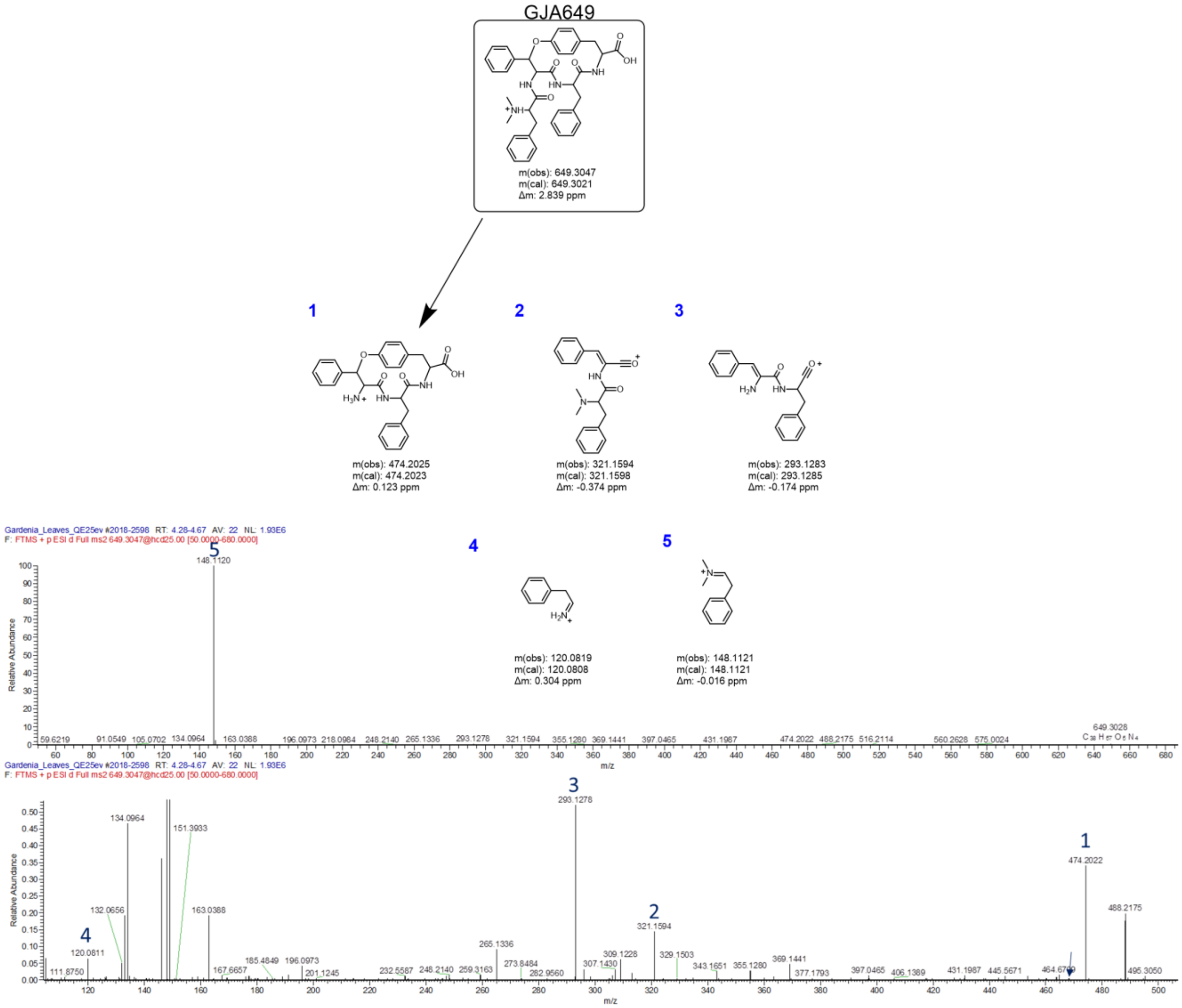

Figure S12. MS/MS spectrum of a feature from *G. jasminoides* extract consistent with a cyclopeptide alkaloid derived from a F-F-F-Y core. The bottom spectrum is a zoom-in of the top spectrum. The mass error is in ppm.

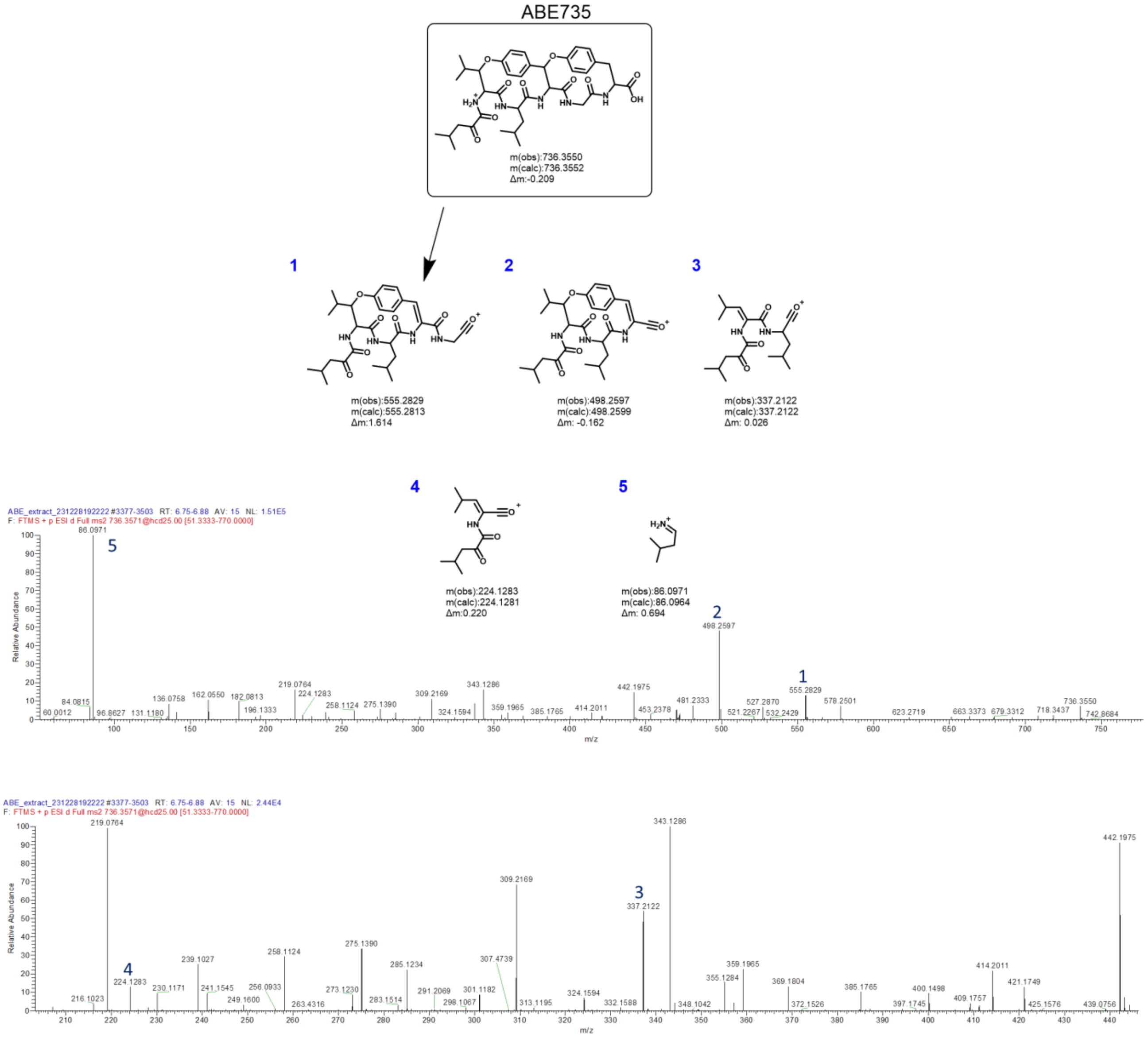

Figure S13. MS/MS spectrum of a feature from *A. bettzickiana* extract consistent with a cyclopeptide alkaloid derived from a L-L-L-Y-G-Y core. The bottom spectrum is a zoom-in of the top spectrum. The mass error is in ppm.

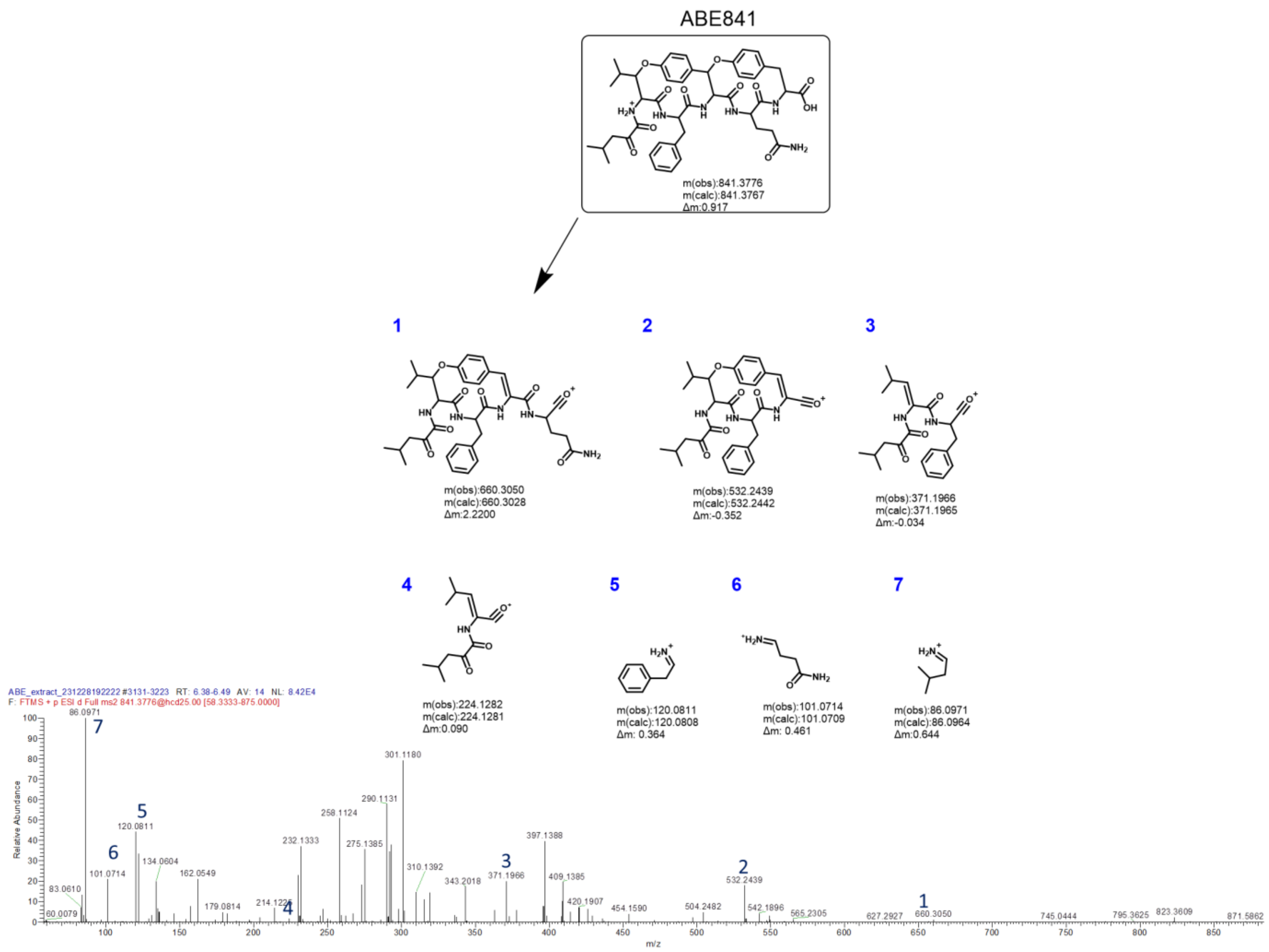

Figure S14. MS/MS spectrum of a feature from *A. bettzickiana* extract consistent with a cyclopeptide alkaloid derived from a L-L-F-Y-Q-Y core. The mass error is in ppm.

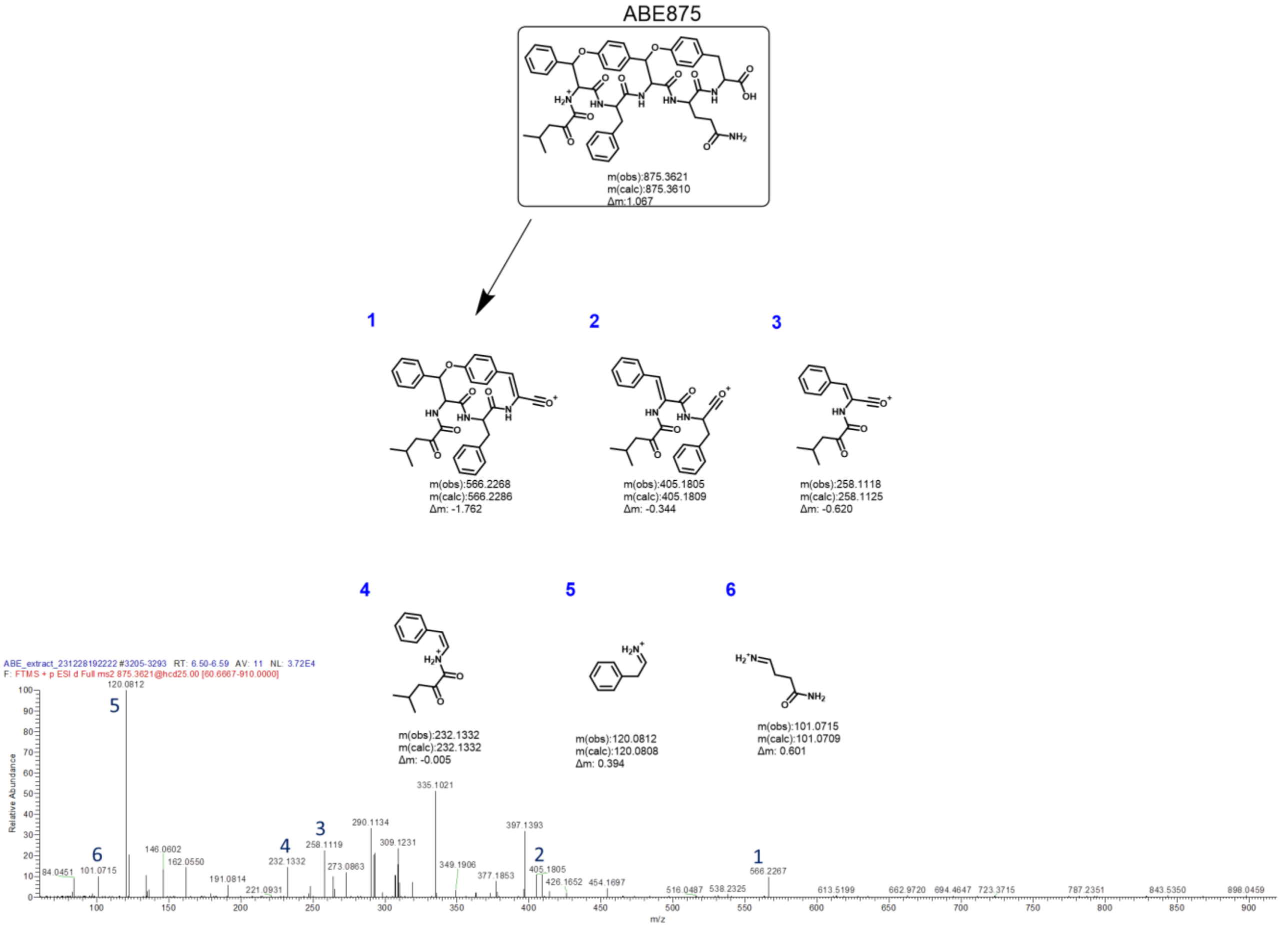

Figure S15. MS/MS spectrum of a feature from *A. bettzickiana* extract consistent with a cyclopeptide alkaloid derived from a L-F-F-Y-Q-Y core. The mass error is in ppm.

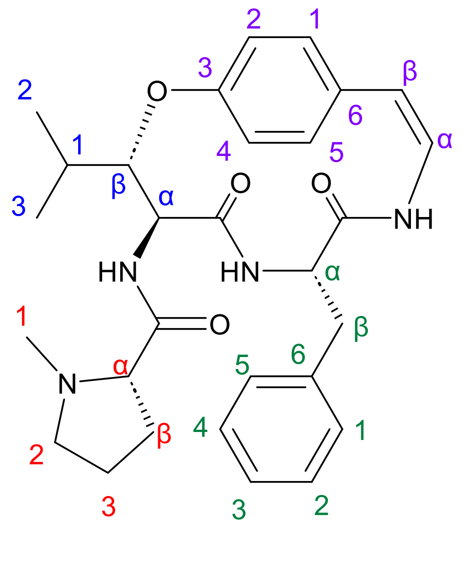

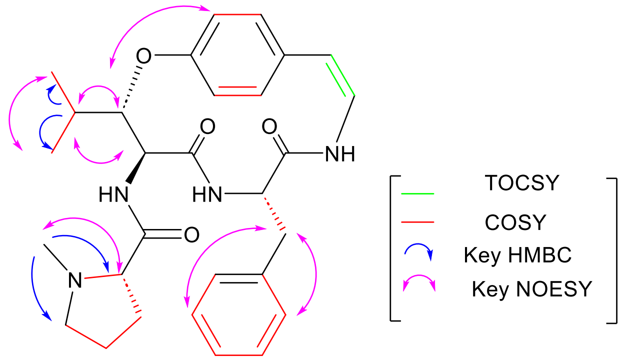

Figure S16. Assigned ceanothine B structure.

**Residue**	**C**	**δ(13C) [ppm]**	**H**	**δ (1 H) (J, d) [ppm]**
Pro	C_α	67.9	H_α	2.74 (4.0, 10.0, dd)
	C_β	30.2	H_β	1.68 (m), 2.16 (m)
	C_1	40.4	H_1	1.97 (s)
	C_2	56.0	H_2	2.33 (m), 3.05(m)
	C_3	23.8	H_3	1.68 (m), 1.79 (m)
Leu	C_α	54.9	H_α	4.42 (8.0, d)
	C_β	80.5	H_β	4.80 (2.0, 8.0, dd)
	C_1	28.8	H_1	1.95(m)
	C_2	13.6	H_2	0.926 (7.0, d)
	C_3	19.2	H_3	1.15 (7.0, d)
Phe	C_α	54.7	H_α	4.22 (m)
	C_β	37.5	H_β	2.59 (12.5, t), 2.85 (13.5, d)
	C_1	129.0	H_1	7.09 (m)
	C_2	128.1	H_2	7.17 (m)
	C_3	126.6	H_3	7.12 (m)
	C_4	128.1	H_4	7.17 (m)
	C_5	129.0	H_5	7.09 (m)
	C_6	136.9	H_6	-
Tyr	C_α	128.0	H_α	6.73 (7.5, d)
	C_β	125.3	H_β	6.19 (7.0, d)
	C_1	130.1	H_1	6.96 (m)
	C_2	117.1	H_2	6.98 (m)
	C_3	156.3	H_3	-
	C_4	120.5	H_4	7.01 (m)
	C_5	129.3	H_5	7.04 (m)
	C_6	130.8	H_6	-

Table S1. NMR assignments of ceanothine B.

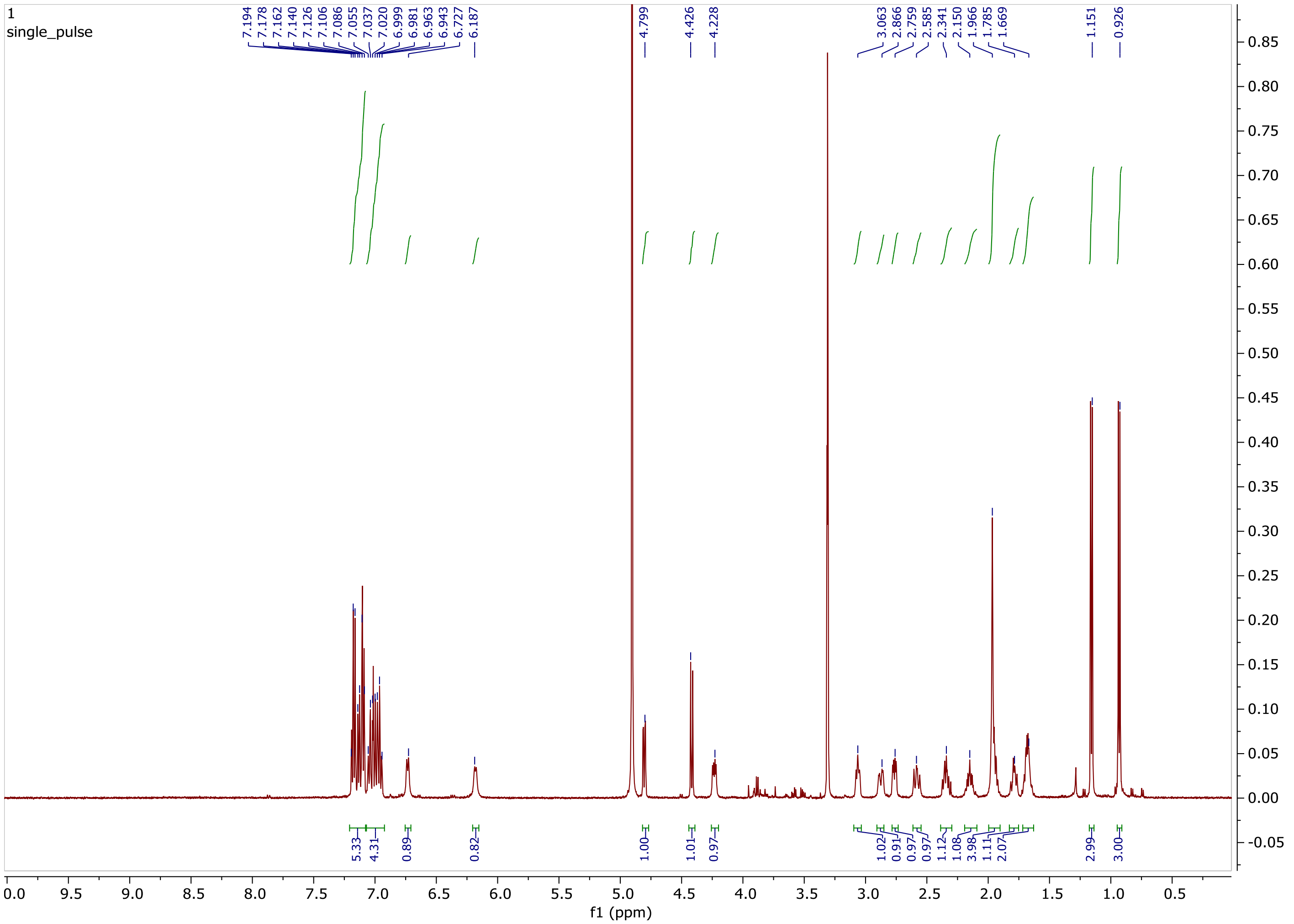

Figure S17. ^{1}H-NMR spectrum of ceanothine B in MeOH-d_4 (500 MHz).

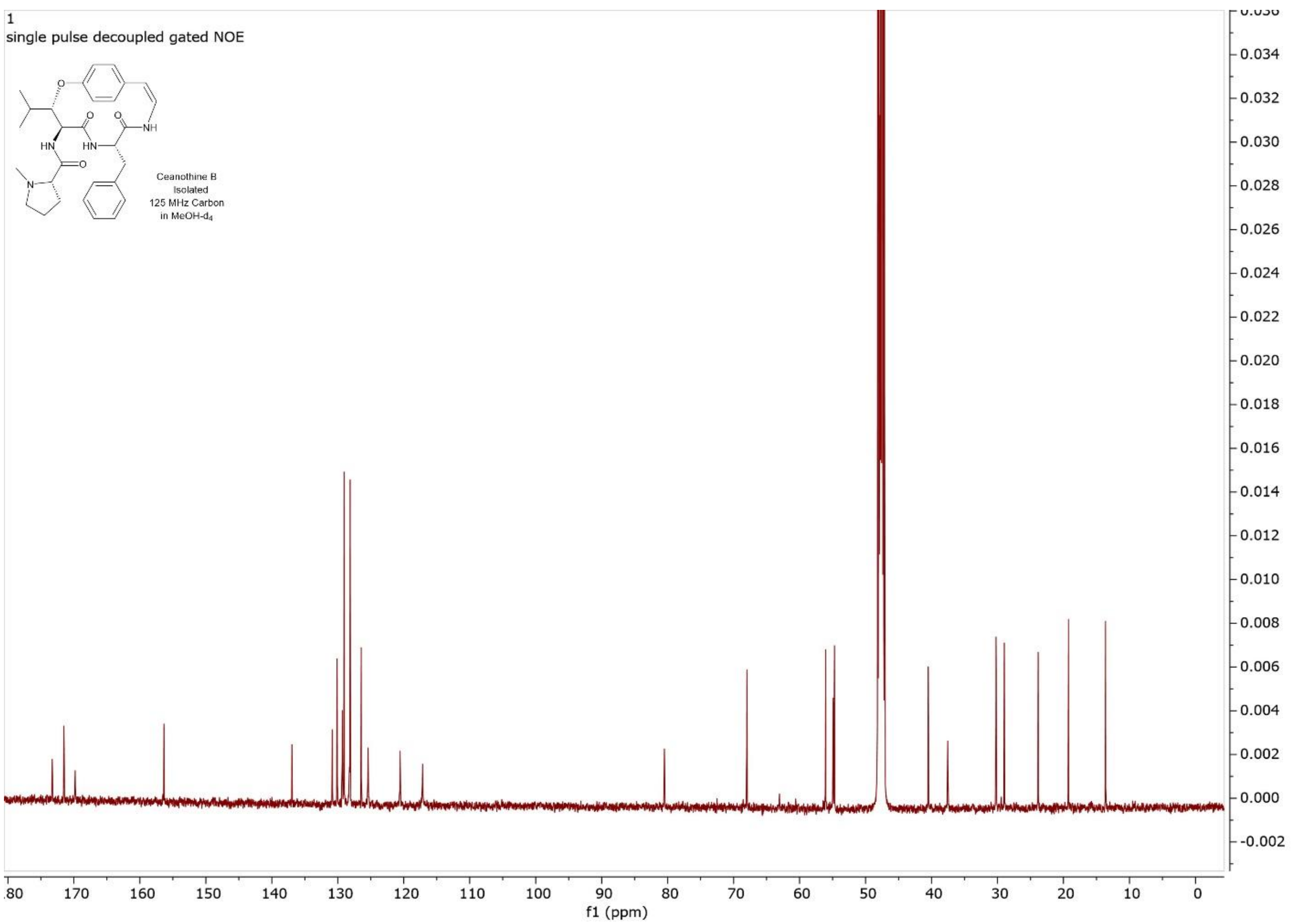

Figure S18. ^{13}C-NMR spectrum of ceanothine B in MeOH-d_4 (500 MHz).

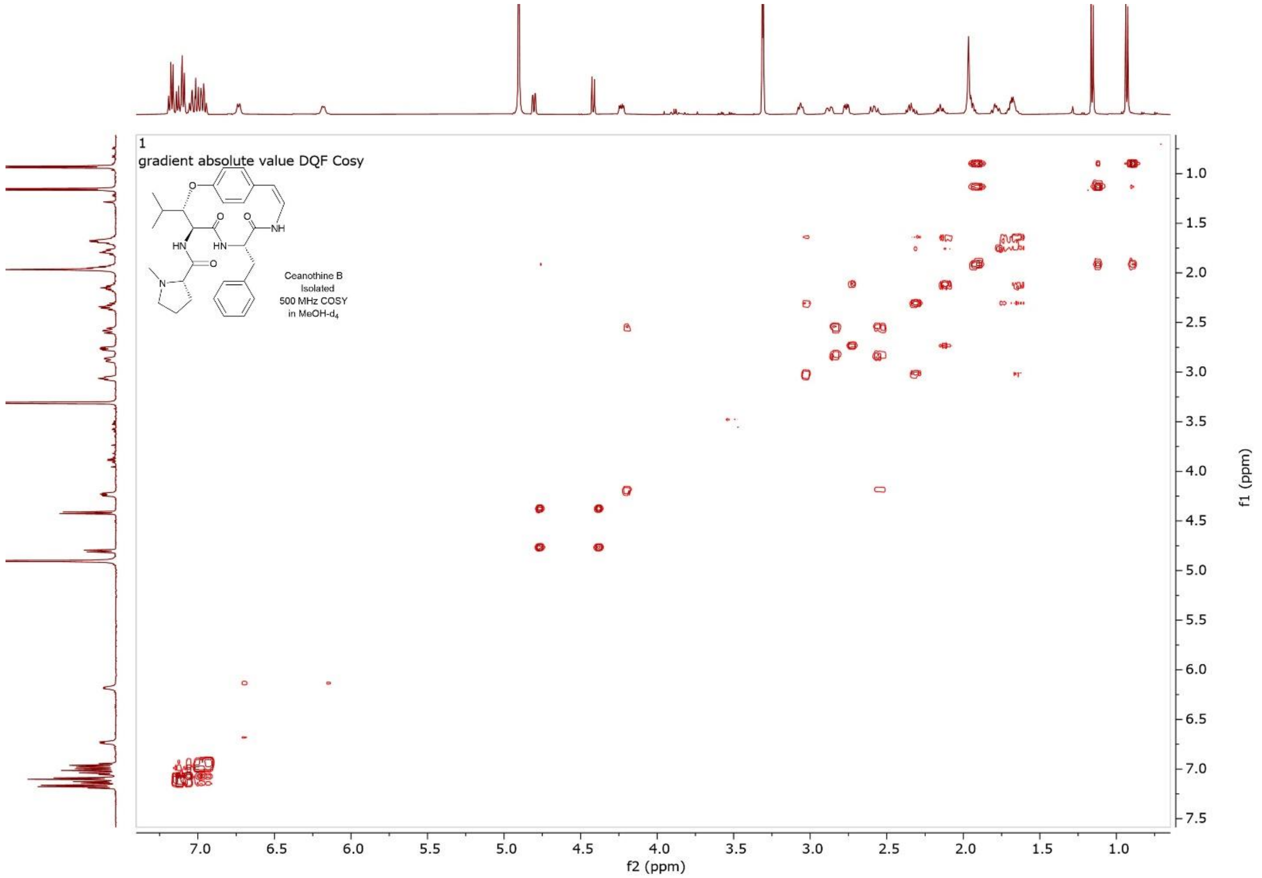

Figure S19. COSY-NMR spectrum of ceanothine B in MeOH-d_4 (500 MHz).

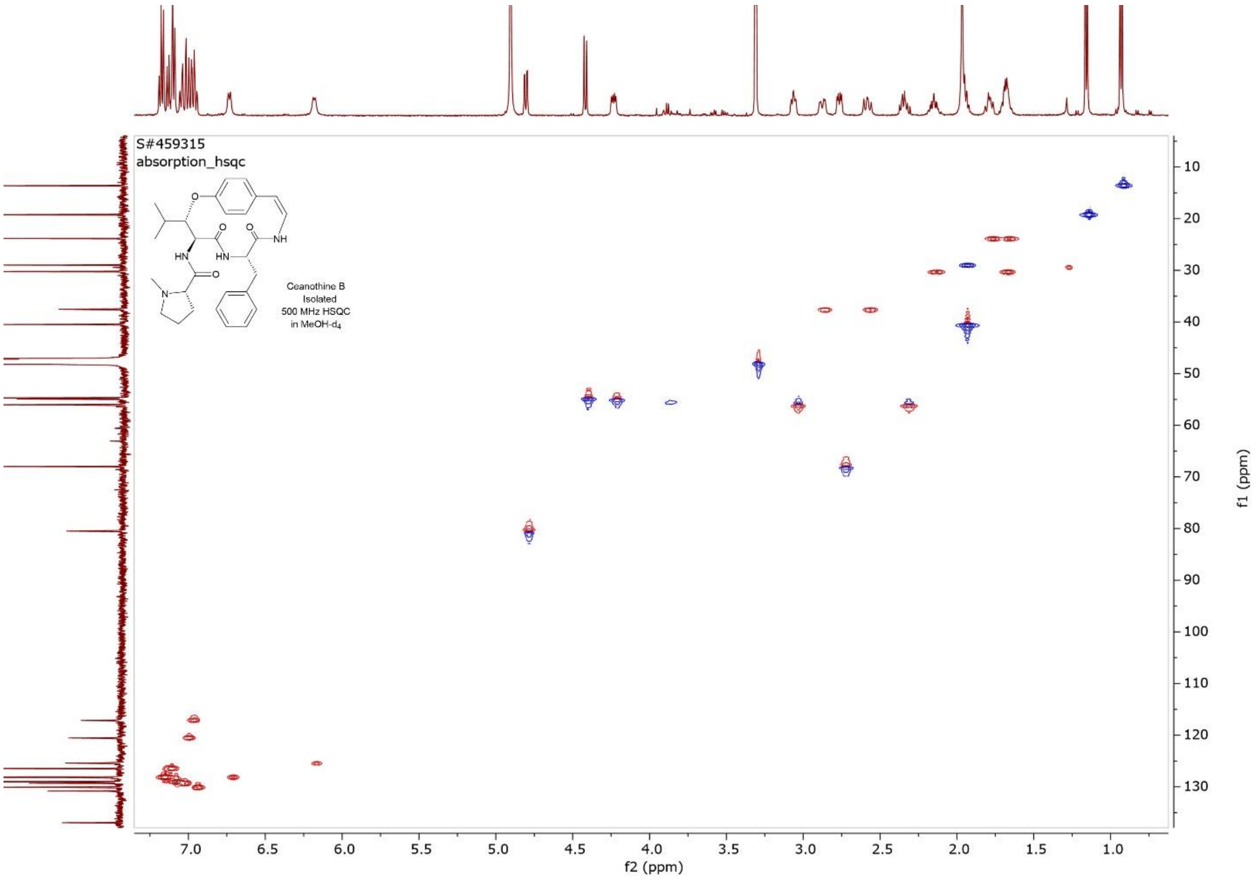

Figure S20. HSQC-NMR spectrum of ceanothine B in MeOH-d_4 (500 MHz).

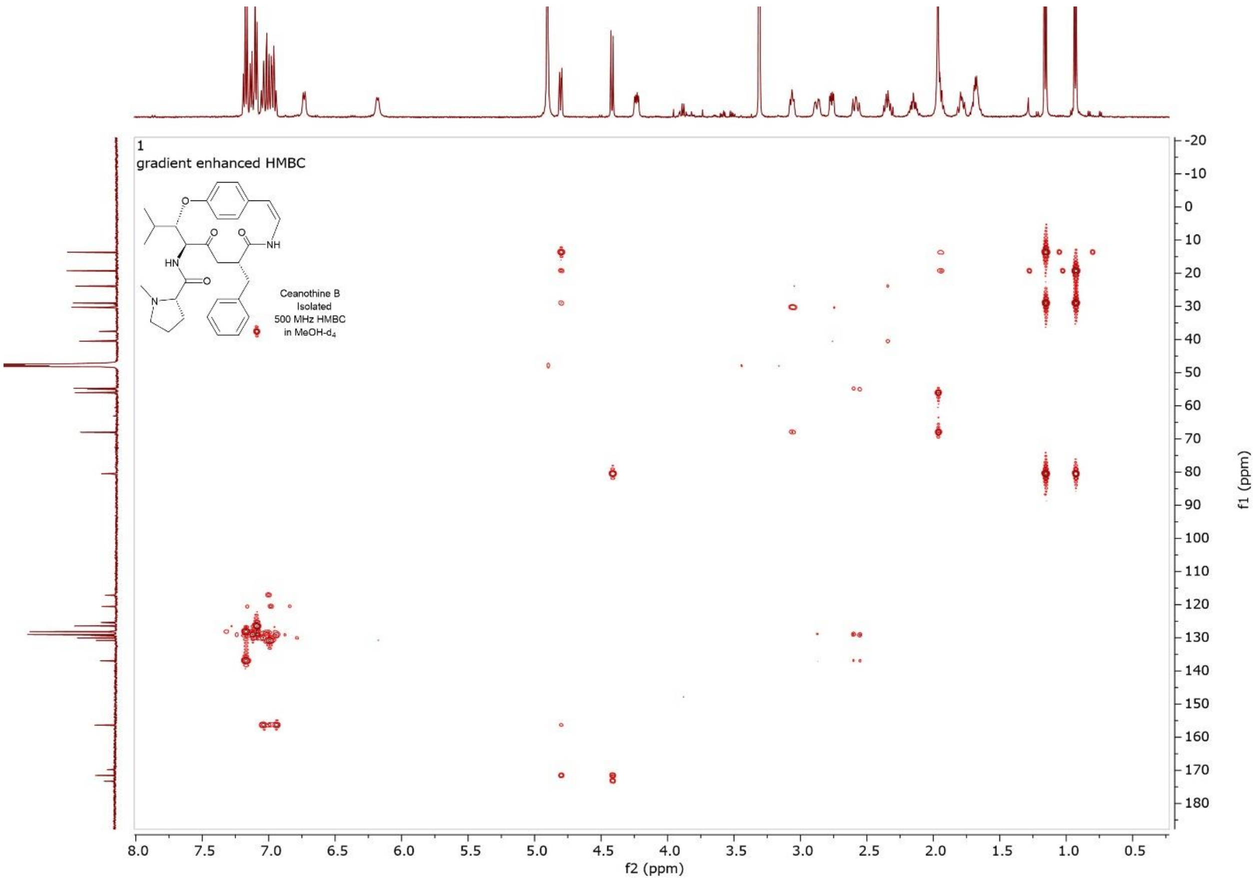

Figure S21. HMBC-NMR spectrum of ceanothine B in MeOH-d_4 (500 MHz).

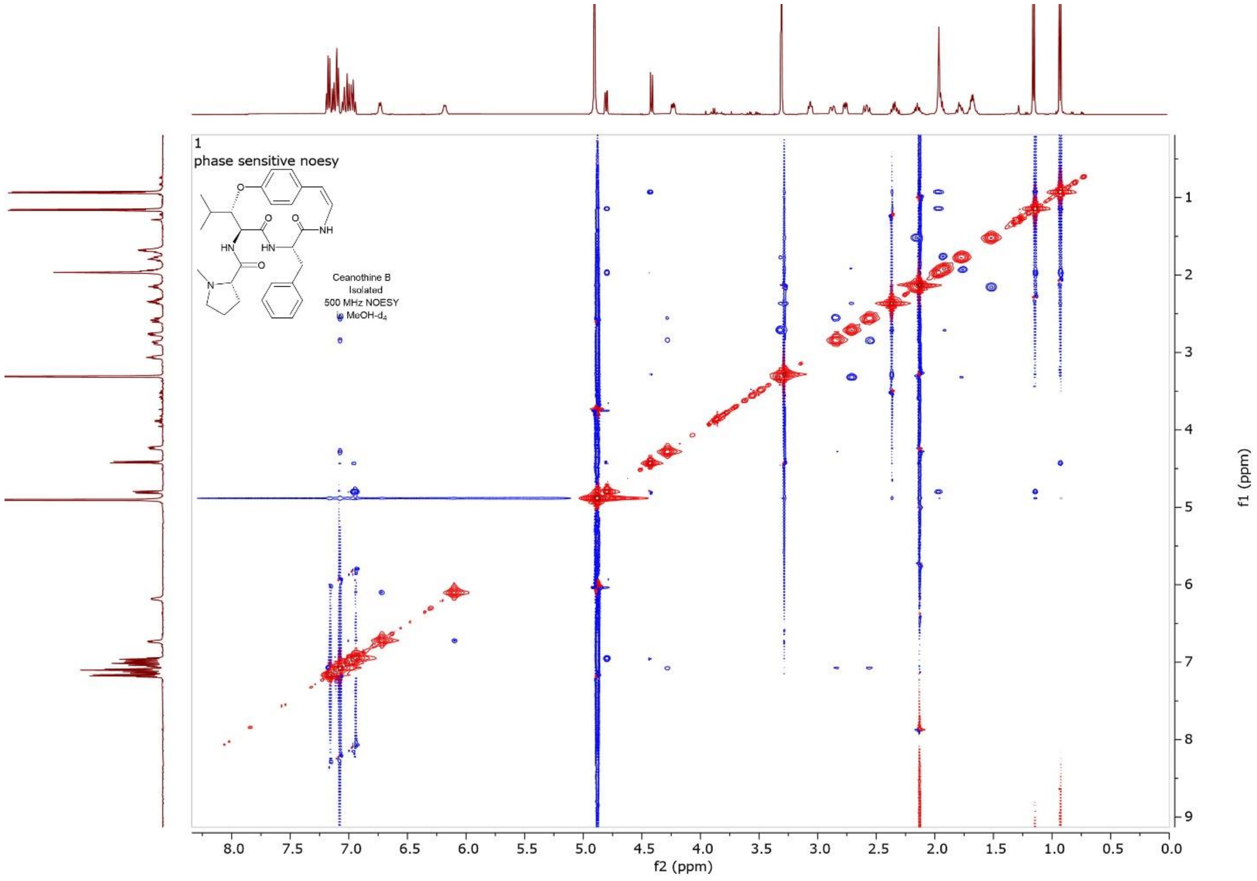

Figure S22. NOESY-NMR spectrum of ceanothine B in MeOH-d_4 (500 MHz).

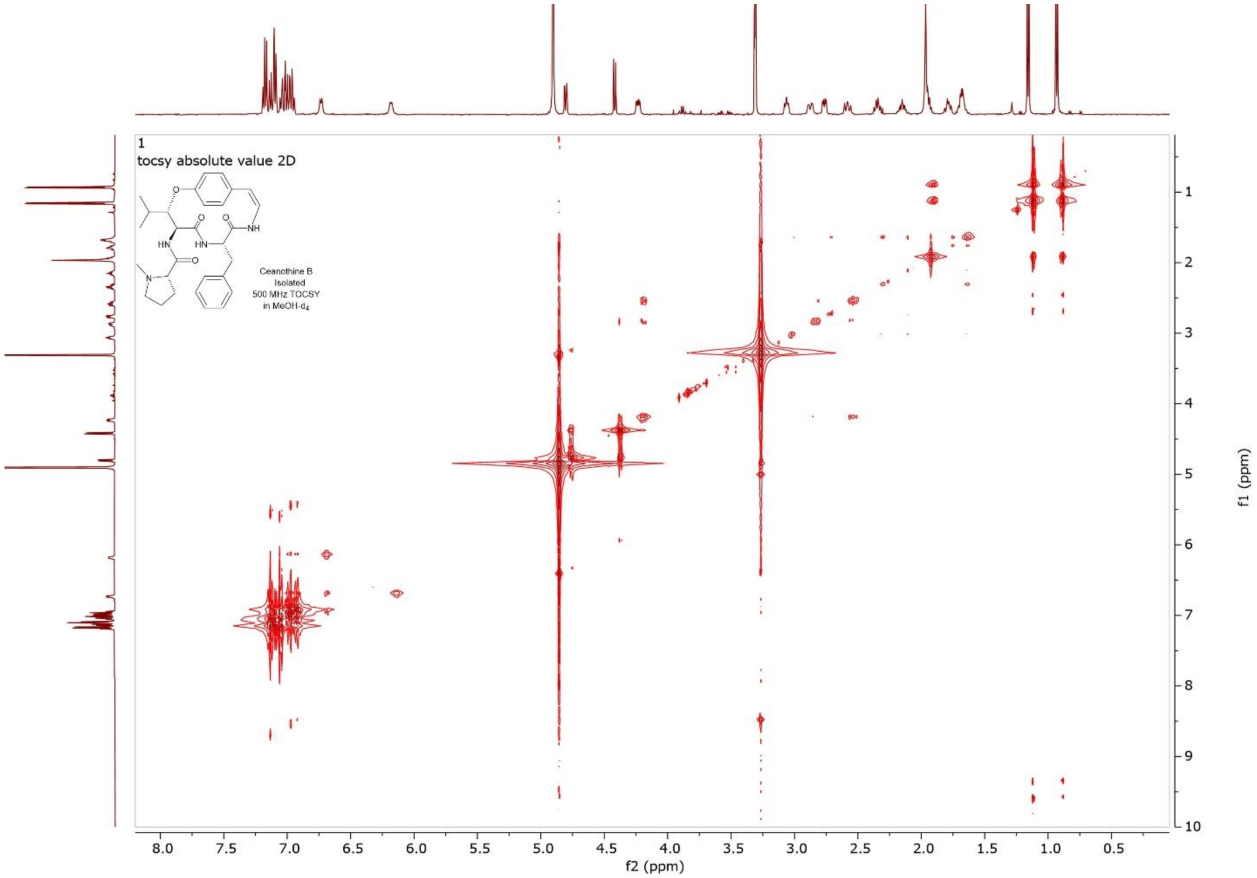

Figure S23. TOCSY-NMR spectrum of ceanothine B in MeOH-d_4 (500 MHz).

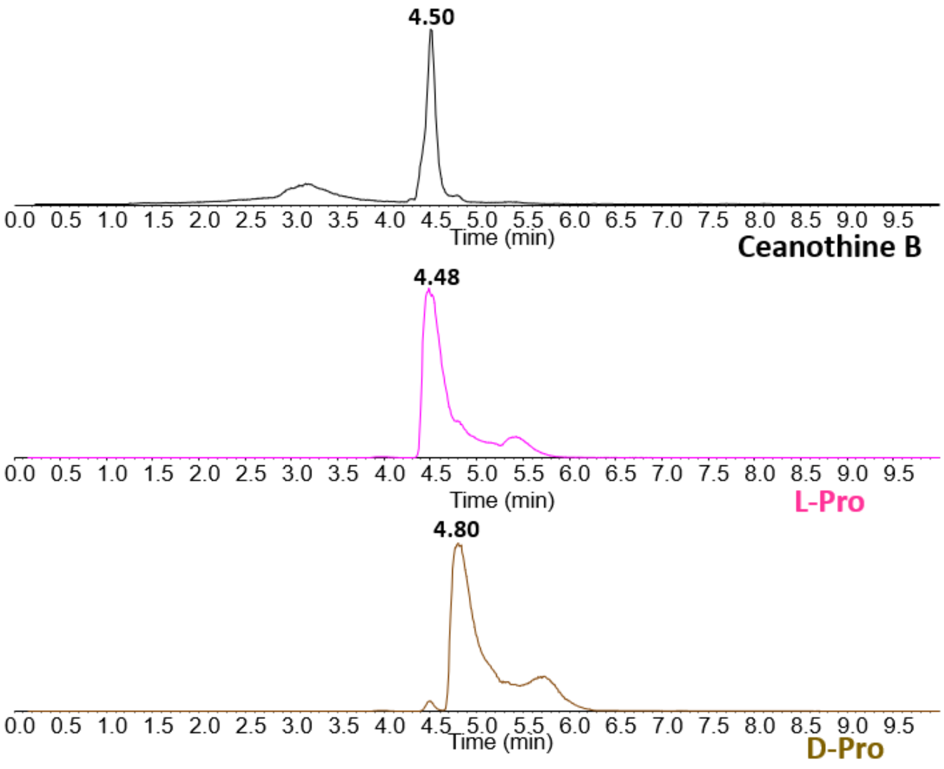

Figure S24: Marfey's analysis for ceanothine B. Positive mode extracted ion chromatogram for hydrolyzed and derivatized ceanothine B sample. Standard amino acid: proline ($[M+H]^+$ 368.12 *m/z*).

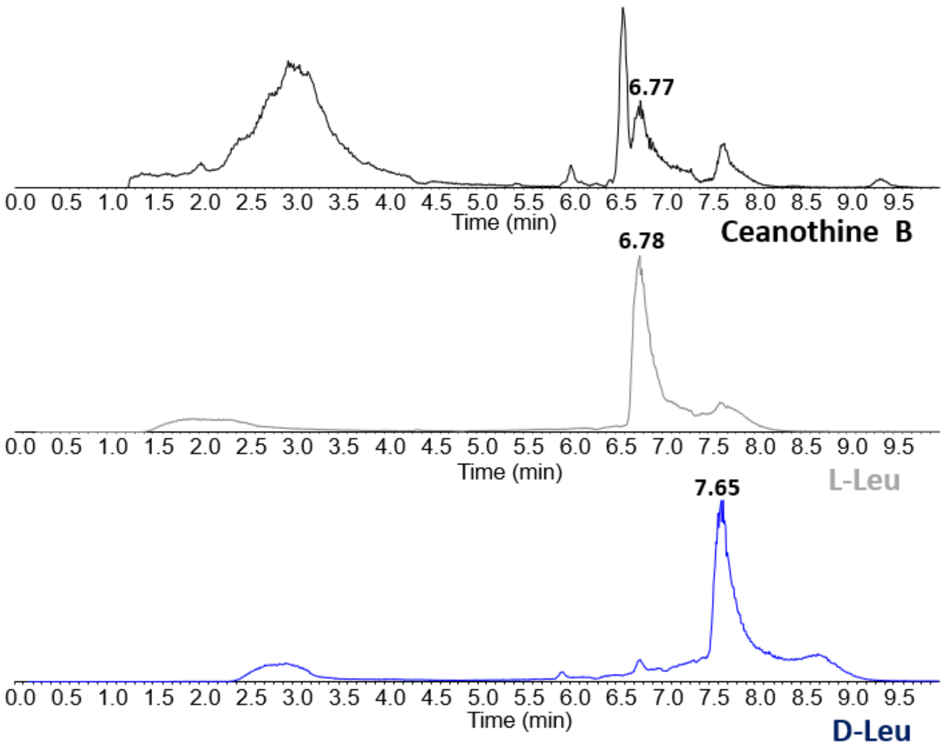

Figure S25. Marfey's analysis for ceanothine B. Positive mode extracted ion chromatogram for hydrolyzed and derivatized ceanothine B sample. Standard amino acid: leucine ($[M+H]^+$ 384.15 *m/z*).

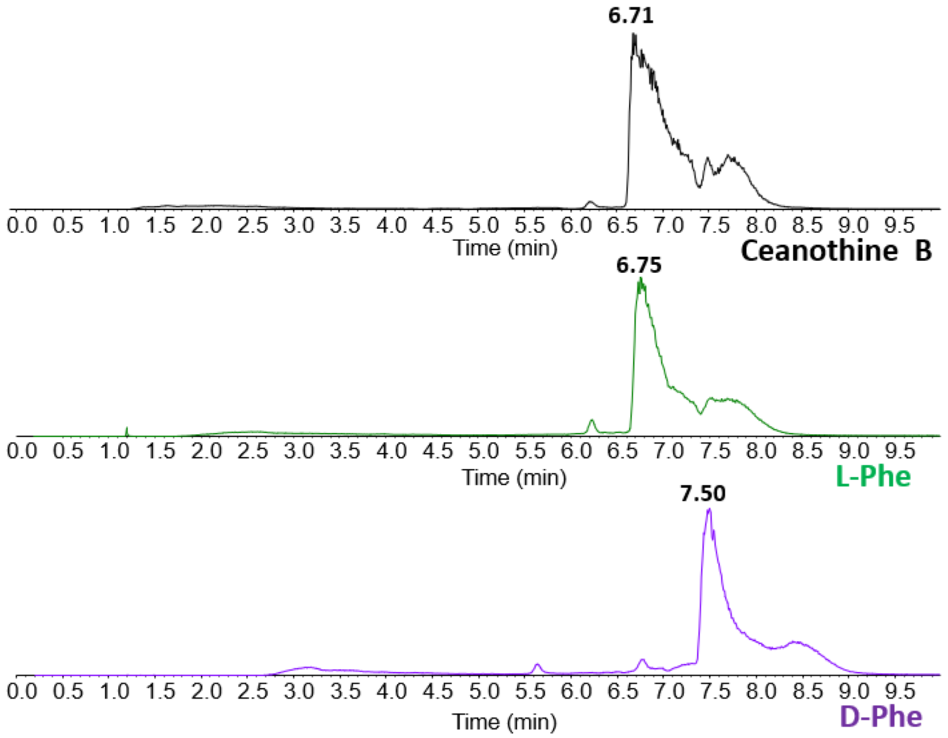

Figure S26. Marfey's analysis for ceanothine B. Positive mode extracted ion chromatogram for hydrolyzed and derivatized ceanothine B sample. Standard amino acid: phenylalanine ($[M+H]^+$ 418.13 *m/z*).

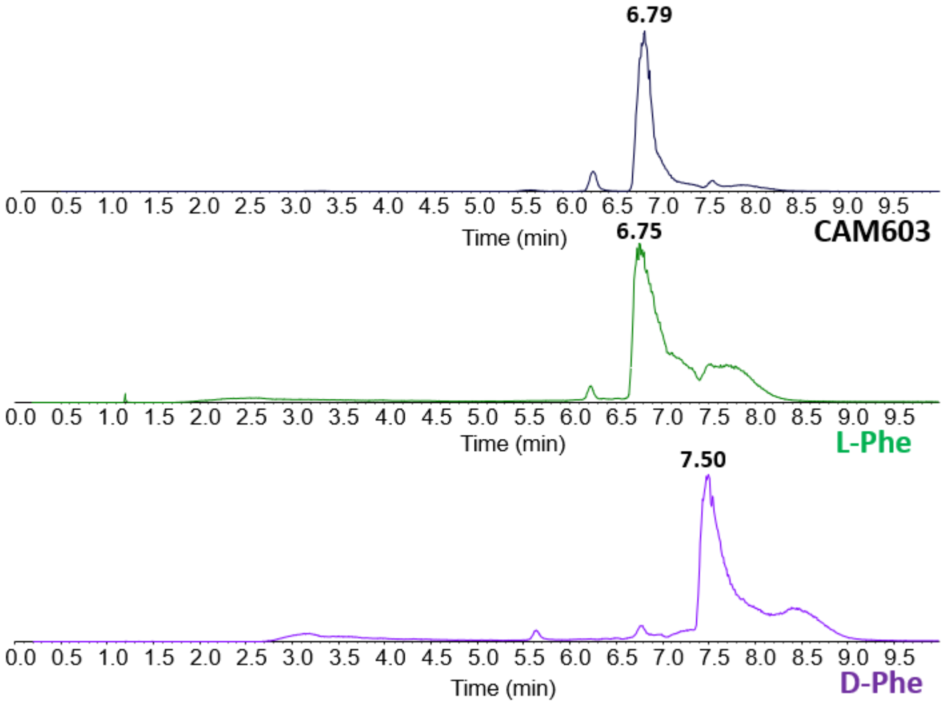

Figure S27. Marfey's analysis for CAM603. Positive mode extracted ion chromatogram for hydrolyzed and derivatized CAM603 sample. Standard amino acid: phenylalanine ($[M+H]^+$ 418.13 *m/z*).

**Experimental**	**Full Scan**
Polarity	Positive
Resolution	35,000
AGC Target	1E6
Maximum IT	50 ms
Scan Range:	300-1000 Da

**Experimental**	**dd-MS^2**
Resolution	17,500
AGC target	1E5
Maximum IT	50 ms
Loop Count (Top N)	5
Isolation Window	1.8 *m/z*
NCE	25

**HESI Source**	
Sheath gas flow rate	48 psi
Aux gas flow rate	11 psi
Sweep gas flow rate	2 psi
Spray voltage (|kV|)	3.00
Spray current (µA)	
Capillary temp. (°C)	256
S-lens RF level	50.0
Aux gas heater temp. (°C):	350

Table S2. Table of the MS data acquisition method parameters.
