# Supplementary material for: Mining raw plant transcriptomic data for new cyclopeptide alkaloids
Source: Beilstein J Org Chem. 2024 Jul 11;20:1548–59. doi: 10.3762/bjoc.20.138 (PMC11250218; doi:10.3762/bjoc.20.138)
Supplement: File 3 — The full cladogram with species names as a high-resolution pdf. [file Beilstein_J_Org_Chem-20-1548-s003.pdf]

BEILSTEIN JOURNAL OF ORGANIC CHEMISTRY

Supporting Information

for

Mining raw plant transcriptomic data for new cyclopeptide alkaloids

Draco Kriger, Michael A. Pasquale, Brigitte G. Ampolini and Jonathan R. Chekan

Beilstein J. Org. Chem. **2024,** *20,* 1548–1559. doi:10.3762/bjoc.20.138

The full cladogram with species names as a high-resolution pdf

License and Terms: This is a supporting information file under the terms of the Creative Commons Attribution License (https://creativecommons.org/licenses/by/4.0). Please note that the reuse, redistribution and reproduction in particular requires that the author(s) and source are credited and that individual graphics may be subject to special legal provisions.
The license is subject to the *Beilstein Journal of Organic Chemistry* terms and conditions: (https://www.beilstein-journals.org/bjoc/terms)

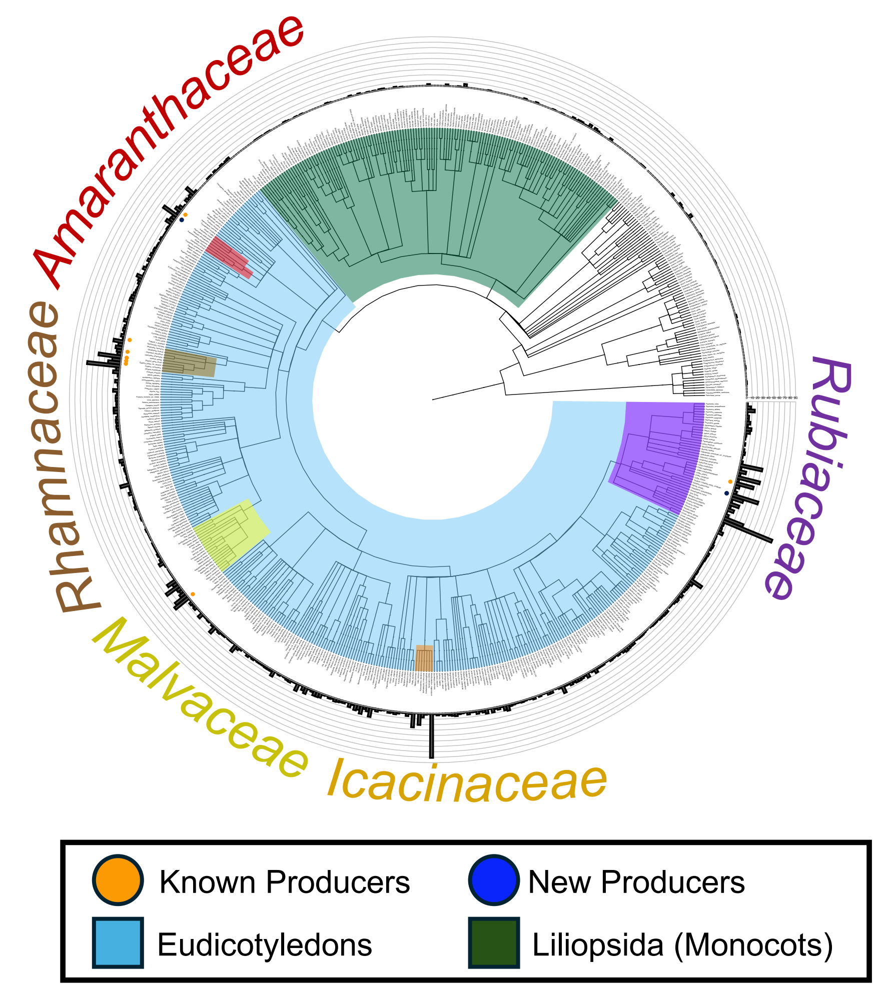

Amaranthaceae
Rhamnaceae
Malvaceae
Icacinaceae
Rubiaceae
Known Producers
New Producers
Eudicotyledons
Liliopsida (Monocots)
